# Experimental and computational investigation on underlying factors promoting high coke resistance in NiCo bimetallic catalysts during dry reforming of methane

**DOI:** 10.1038/s41598-020-80287-0

**Published:** 2021-01-12

**Authors:** Tinnakorn Saelee, Mongkol Lerdpongsiripaisarn, Meena Rittiruam, Siriwimol Somdee, Anchittha Liu, Supareak Praserthdam, Piyasan Praserthdam

**Affiliations:** 1 High-Performance Computing Unit (CECC-HCU), Center of Excellence On Catalysis and Catalytic Reaction Engineering (CECC), Chulalongkorn University, Bangkok, 10330 Thailand; 2 Center of Excellence On Catalysis and Catalytic Reaction Engineering (CECC), Chulalongkorn University, Bangkok, 10330 Thailand

**Keywords:** Chemical engineering, Natural gas, Climate-change mitigation, Chemical engineering, Environmental chemistry, Catalysis, Heterogeneous catalysis

## Abstract

Global warming remains one of the greatest challenges. One of the most prominent solutions is to close the carbon cycle by utilizing the greenhouse gas: CO_2,_ and CH_4_, as a feedstock via the dry reforming of methane (DRM). This work provided an insight into how the NiCo bimetallic catalyst can perform with high stability against coking during DRM compared to the Ni and Co monometallic catalysts, in which the experimental and computational techniques based on density functional theory were performed. It was found that the high stability against coking found on the NiCo surface can be summarized into two key factors: (1) the role of Co weakening the bond between a Ni active site and coke (2) significantly high surface coke diffusion rate on NiCo. Moreover, the calculation of the surface fraction weighted rate of coke diffusion which modeled the real NiCo particle into four regions: Ni-dominant, Co-dominant, NiCo-dominant, and the mixed region consisting a comparable amount of the former there regions, have shown that the synthesis of a NiCo particle should be dominated with NiCo region while keeping the Ni-dominant, and Co-dominant regions to be as low as possible to facilitate coke diffusion and removal. Thus, to effectively utilize the coke-resistant property of NiCo catalyst for DRM, one should together combine its high coke diffusion rate with coke removal mechanisms such as oxidation or hydrogenation, especially at the final diffusion site, to ensure that there will not be enough coke at the final site that will cause back-diffusion.

## Introduction

The dry reforming of methane (DRM) is a promising process towards the utilization of two main greenhouse gases (GHGs) causing global warming: carbon dioxide (CO_2_) and methane (CH_4_), while producing syngas: hydrogen (H_2_) and carbon monoxide (CO) useful as a feedstock for value-added chemical productions^1–4^. Regarding the DRM catalyst, Nickel-based catalysts are mostly selected for the DRM process due to high activity and low price^5–10^. However, the main challenge to put DRM into practical use is the deactivation due to coking of the catalysts used in the process^11–15^. Alternative choices other than nickel: the noble metals, e.g., rhodium (Rh), ruthenium (Ru), and palladium (Pd), providing higher DRM activity and better stability are promising candidates; they are too expensive, making the process impractical^16,17^. Therefore, an alternative solution is to utilize the alloy of Ni, which help resist coking as well as to facilitate coke removal on the catalyst as various studies showed that incorporating Co into Ni forming a NiCo alloy catalyst enhanced DRM activity and stability than that of Ni catalysts with lower price than the noble metals^18–23^. So, the understanding of the promotional effects of Co in the bimetallic systems is vital for the design of reactive-stable DRM catalysts. Thus, in general, the experimental characterizations are applied to track the changes in catalyst surface characteristics, e.g., oxidation states, coke accumulation before and after the reaction, while the reaction determined how catalysts perform in terms of activity and stability^24,25^. Besides, in terms of computational characterizations, the role of Co on NiCo catalyst should be well investigated in terms of electronic properties of catalyst surface as well as the characteristics of an active site for the use in catalyst design. Therefore, the computational techniques based on the density functional theory (DFT), one of the highly efficient methods in terms of computational time and reliability, are suitable for such investigation^26–29^. Recently, Tu et al.^30^ provided an extensive experimental and computational investigation on an improved DRM activity and stability of NiCo due to reactive oxygen species on metal active sites. They found that the presence of reactive oxygen species promotes carbonaceous intermediates (CH_x_^*^) removal during DRM resulting in high availability of the active sites for C–H bond activation. However, the insight into how the two metals in NiCo catalyst: Ni and Co interact and provide high coke resistance is still in need. Previously, we successfully applied DFT with microkinetic modeling to evaluate the reactivity and stability of the Ni–Ni_3_C–NiO catalyst use in DRM and revealed that enough amount of NiO phase is needed to avoid high coke formation together with the control of Ni_3_C phase to be lower than 10% of the total catalyst surface^31–33^. In this work, we applied experimental characterizations and reaction testing combined with the DFT analysis to investigate the insight effect of NiCo catalyst surface in terms of coke behavior and coke resistance. The descriptor for stability-testing included (1) interactions between coke and catalyst surfaces, (2) the electronic properties reflected through Bader charge analysis in which the effects of different coke species size were also studied, and (3) the mobility of coke from the strongest adsorption site to a nearby site on pure Ni, Co, and NiCo surfaces. Also, the coke model for coke diffusion is proposed through the 3-stage coke diffusion, which can describe the coke-resistant surface. Besides, the coke mobility during the 3-stage diffusion is modeled on a real NiCo particle as a function of Ni-dominant, Co-dominant, and NiCo-dominant regions described by the ternary contour plot of coke diffusion rate on the surface.

## Results and discussion

### Effects of Co on catalytic activity and coke-resistance during DRM

The performance of DRM catalysts is evaluated based on their activity in terms of the product yield, H_2_/CO ratio, and their stability in terms of coke deposition at DRM reaction conditions summarized in Table 1. It was found that the Ni monometallic and Co catalysts exhibited similar CO yield at 18.48% and 15.21%, respectively, but the Ni catalyst yield higher H_2_ than that of the Co at 30.40% and 10.63%, respectively. This suggested a better activity of Ni than the Co catalyst. However, in the aspect of stability, coke accumulation derived via the thermogravimetric analysis (TGA) profiles on Ni is found to be significantly higher than that on the Co catalyst as depicted in Fig. S1. This indicated lower resistance of Ni to coking than Co, which is a common problem found in Ni DRM catalysts^34–36^.

**Table 1:** The coke accumulation on spent Ni, NiCo of different Ni:Co ratio, and Co catalysts used in the DRM process.

Catalyst	Co content (wt%)	CO yield (%)	H_2_ yield (%)	H_2_/CO ratio	Amount of coke (%)	coke mitigation from pure Ni ^a^
Ni	0	18.48	30.40	1.6	74.15	0.00
NiCo-70:30	30	25.48	45.11	1.8	65.01	9.14
NiCo-50:50	50	22.50	38.09	1.7	53.52	20.63
Co	100	15.21	10.63	0.70	4.84	69.31

^a^Coke mitigation is calculated from the changes in coke deposition compared to pure Ni catalyst.

Therefore, we performed a study to understand how NiCo bimetallic catalysts have combined high activity of Ni with high coke-resistance of Co. Alloying Co into Ni forming NiCo catalysts resulted in an increase in CO and H_2_ yield promoting activity while provided a high H_2_/CO ratio, like in the case of Ni monometallic catalysts. Considering coking, which reflected stability, NiCo catalysts reduced coke accumulation up to 20% compared to a Ni monometallic catalyst confirmed by the TGA profiles, where this lower coke deposition was found at higher Co loading, which was also confirmed by our previous study^37^.

As a result, to understand these combined promotional effects of Ni and Co in NiCo bimetallic catalysts promoting activity and stability for the design of high-performance DRM catalysts computational techniques based on density functional theory (DFT) was performed, where the NiCo-50:50 was chosen as a model of study since it exhibited high H_2_/CO ratio comparable to Ni with enhanced stability from low coke deposition.

### Catalyst model construction and morphological analysis

**Figure 1:**
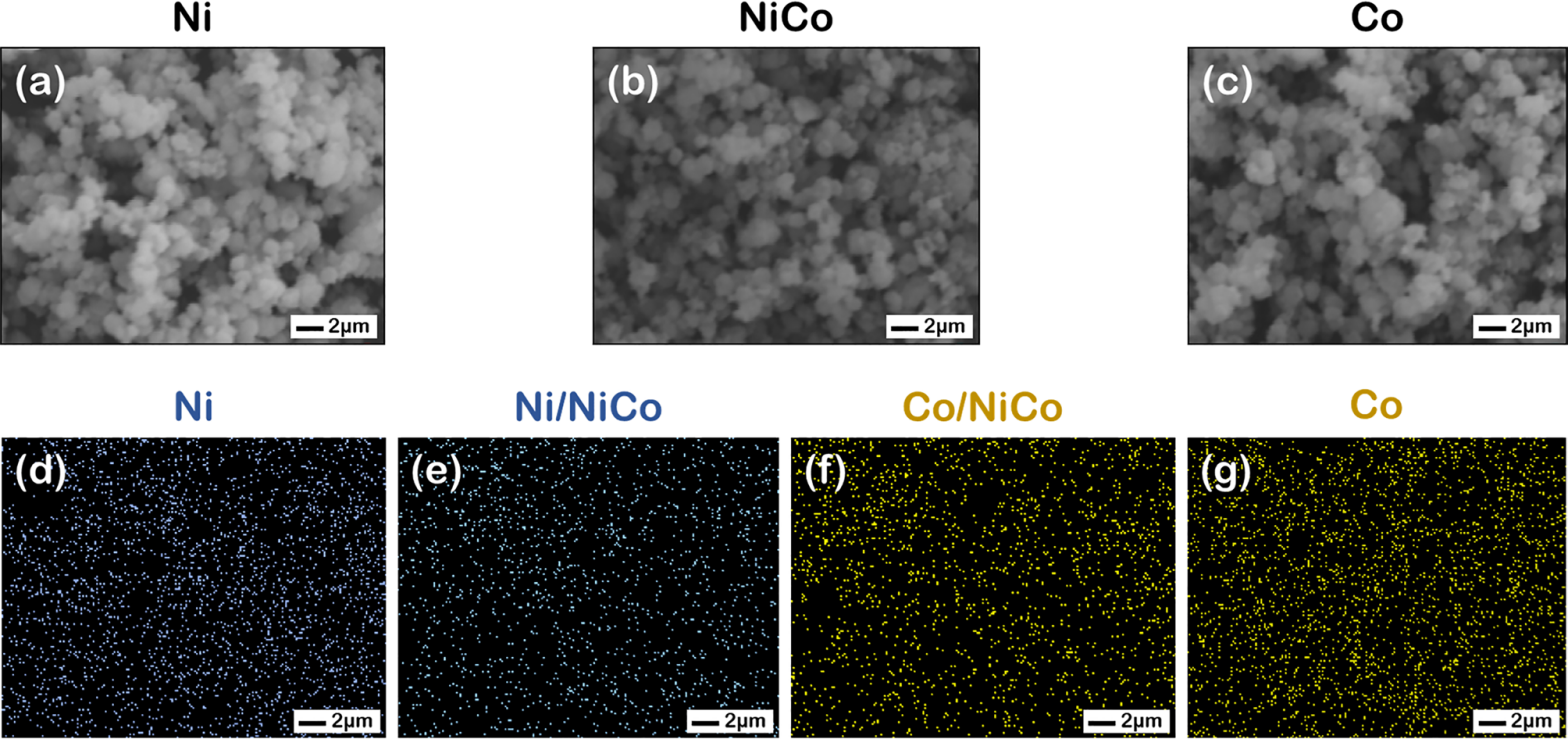
SEM profiles of (**a**) Ni catalyst, (**b**) NiCo-50:50 NiCo catalyst, and (**c**) Co catalyst and SEM–EDX profiles of (**d**) Ni elemental mapping in Ni catalyst, (**e**) Ni and (**f**) Co elemental mapping of NiCo-50:50 NiCo catalyst, and (**g**) Co elemental mapping in the Co catalyst. Note that the blue and yellow dots represent Ni and Co elemental mapping, respectively.

**Figure 2:**
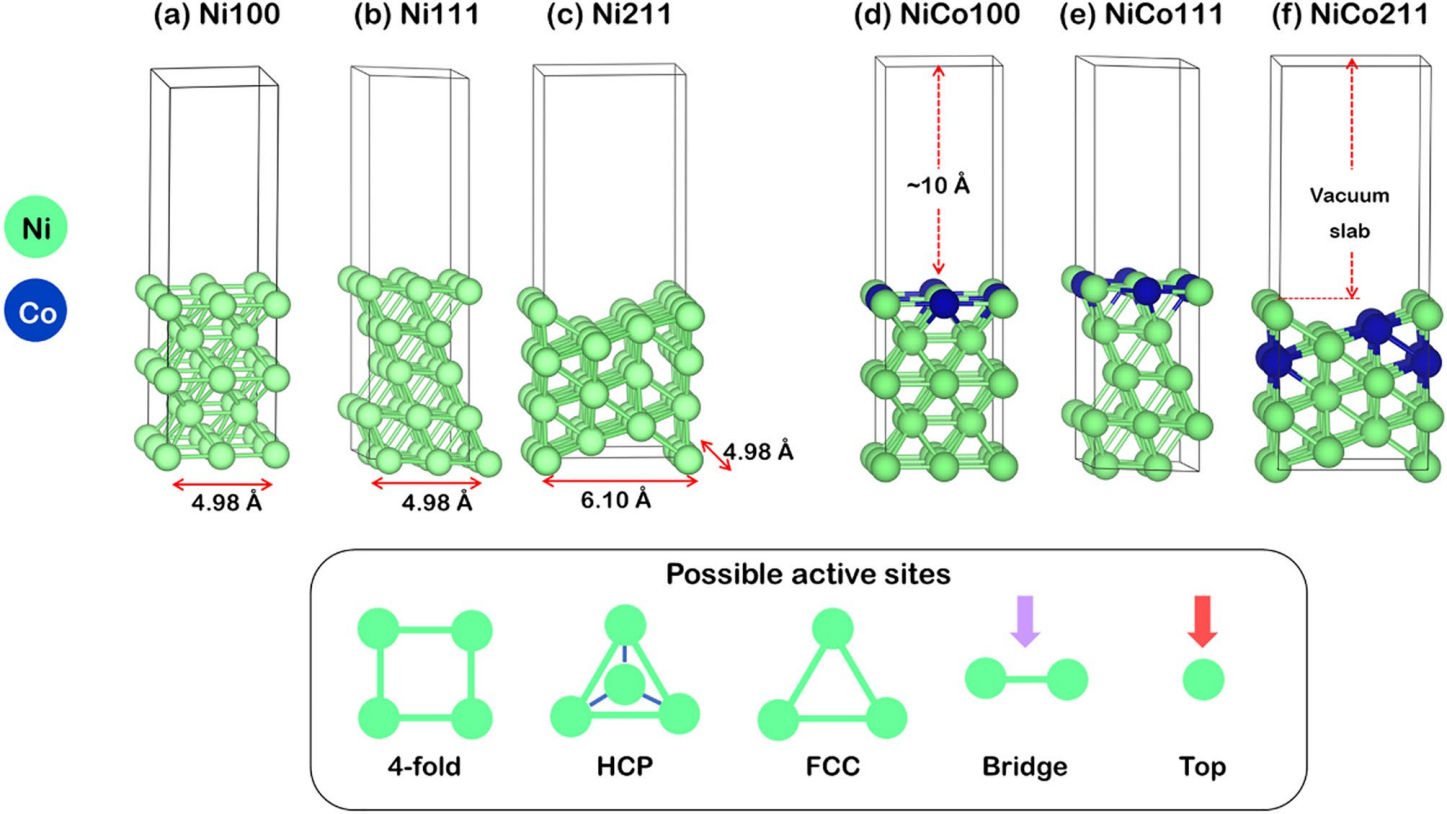
Surface slab models of (**a**) Ni100, (**b**) Ni111, (**c**) Ni211, (**d**) NiCo100, (**e**) NiCo111, and (**f**) NiCo211, where and possible active sites are fourfold, HCP, FCC, Bridge, and Top sites.

As the NiCo-50:50 was selected as a model of study, the morphological data of the Ni and NiCo catalysts with Ni and Co elemental mapping analyzed by SEM–EDX shown in Fig. 1 were used as the initial information for model construction. The SEM–EDX profiles of NiCo suggested that both Ni and Co metals were well distributed on the catalyst surface, in which large clusters of either Ni or Co was not observed. Thus, based on the data, the model of alternately arranged Ni and Co atoms for various facets were constructed. Besides, all surface slab models and their active sites are shown in Fig. 2(a-2), (b-2), and (c-2).

For slab models, the 3 × 3 supercells for Ni100, Ni111, and Co001 flat surfaces and the 3 × 4 supercell for Ni211 stepped surface were constructed, depicted in Fig. 2, while the slab model of Co001 surface is shown in Fig. S1 in the supplementary document. All models comprised a 10-Å vacuum region along the z-axis to avoid interactions from the periodicity of the slab. The NiCo100 and NiCo111 flat surfaces were constructed by substituting two Ni atoms on the Ni flat surface with Co atoms, while the NiCo211 stepped surface was constructed by substituting four Ni atoms with Co atoms. These models represented the NiCo with a Ni:Co ratio of 50:50. During the optimization, three and one bottom layers were fixed to their bulk lattice parameter in flat and stepped surfaces, respectively, while the top two layers and adsorbed species are allowed to relax. All possible active sites on the Ni100, Ni111, and Ni211 slabs are illustrated in Fig. 2(a-1) to (c-1), while the NiCo100, NiCo111, and NiCo211 were shown in Fig. 2(a-2) to (c-2).

### Surface electronic properties of fresh catalysts

**Figure 3:**
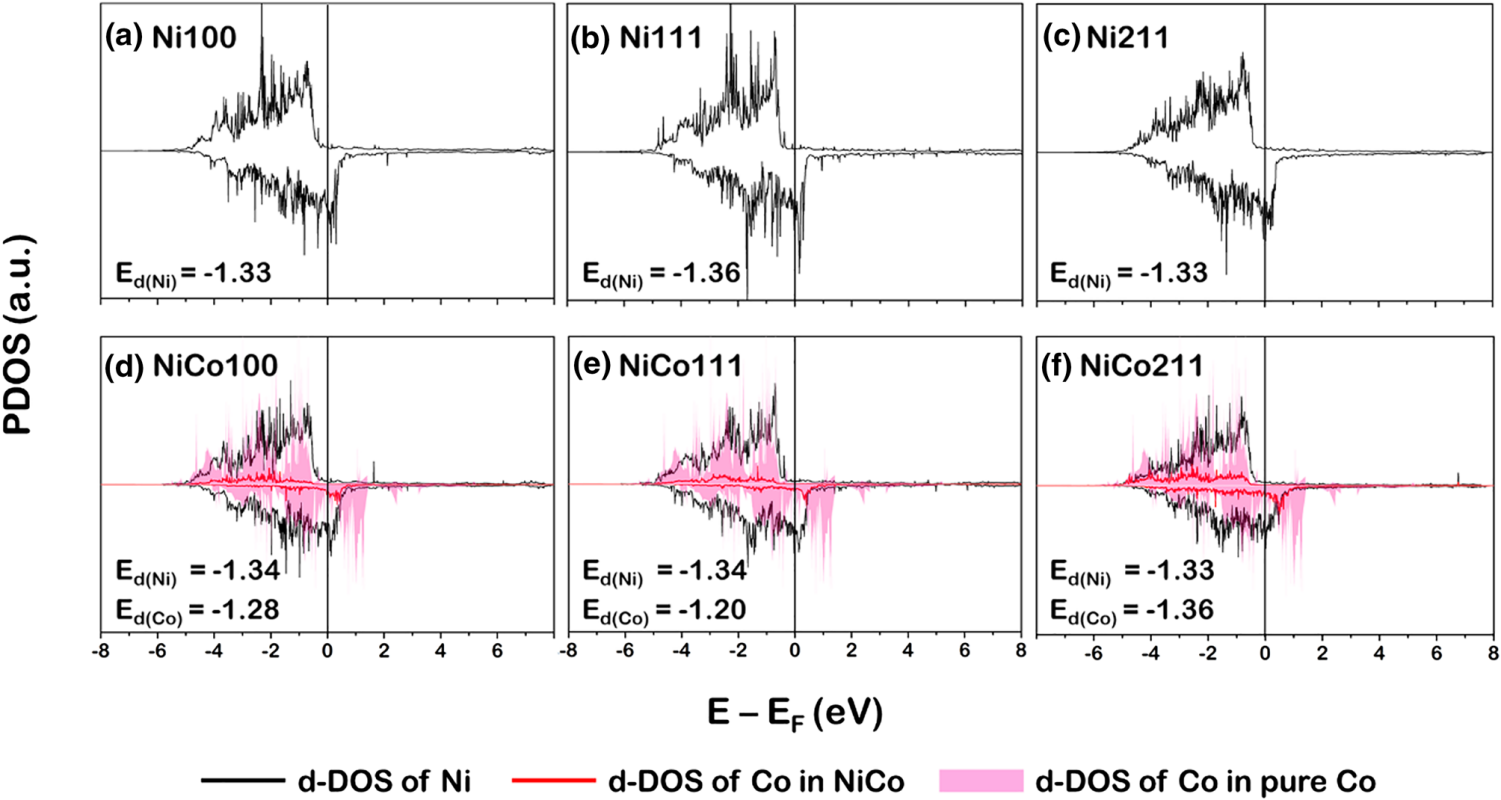
The d-state PDOS of the Ni for (**a**) Ni100, (**b**) Ni111, (**c**) Ni211, and Ni and Co for (**d**) NiCo100, (**e**) NiCo111, and (**f**) NiCo211 surfaces.

Having modeled and optimized the Ni and NiCo catalyst surfaces, the electronic properties of these fresh catalysts are investigated. The projected density of state (PDOS) profiles of d-orbital (d-PDOS) plotted in Fig. 3 for Ni100, Ni111, Ni211, Co001, NiCo100, NiCo111, and NiCo211 facets were analyzed. The PDOS can reflect the activity of the catalyst based on the analysis of bonding and antibonding states located at the lower (left side of the E_F_) and higher (right side of the E_F_) regions, respectively, and separated by the Fermi level (at E_F_ = 0 eV).

The asymmetrical d-PDOS of Ni (Ni-d-PDOS represented by a black line in Fig. 3) and Co (Co-d-PDOS represented by a pink filled region in Fig. 3) indicated the common ferromagnetic characteristics for such system^28,38^. It was observed that each Ni-d-PDOS profile of Ni100, Ni111, and Ni211 surfaces still retained its shape after doping with Co to form NiCo. This can be implied that introducing Co active sites did not reduce the activity compared to the Ni monometallic catalyst. In addition, the activity of the catalyst surface can also be determined by the d-band center (E_d_) proposed by Nørskov et al.^39,40^, which can be calculated as follows. Note that the parameter ρ_d_ represented the d-PDOS.

1 $${\text{E}}_{{\text{d}}} = \frac{{\mathop \smallint \nolimits_{ - \infty }^{\infty } {\text{E}}\rho_{{\text{d}}} ({\text{E}} - {\text{E}}_{{\text{F}}} ){\text{dE}}}}{{\mathop \smallint \nolimits_{ - \infty }^{\infty } \rho_{{\text{d}}} ({\text{E}} - {\text{E}}_{{\text{F}}} ){\text{dE}}}}$$

Considering various facets of Ni monometallic catalyst, the E_d_ values are − 1.33 eV for Ni100, − 1.36 eV for Ni111, and − 1.33 eV for Ni211. Comparing to the NiCo bimetallic surface, they showed similar E_d_ values to the Ni monometallic ones, where Ni-element E_d_ values are − 1.34 eV for NiCo100, − 1.35 eV for NiCo111, and − 1.33 eV for NiCo211. Thus, doping with Co did not affect the catalytic activity of the NiCo catalyst suggested by similar values of E_d_ of Ni monometallic and NiCo bimetallic surfaces, which is in good agreement with our DRM reaction testing showing comparable CO and H_2_ yields and H_2_/CO ratio.

### Surface behavior to coke on Ni and NiCo surfaces

Another aspect of the performance evaluation is the stability against coke formation. Hence, we modeled the situation during coke deposition on the catalyst surface via the carbon atom adsorption on various facets of Ni and NiCo surfaces to help understand the behavior of coke to surface during deactivation on Ni and NiCo catalysts.

**Figure 4:**
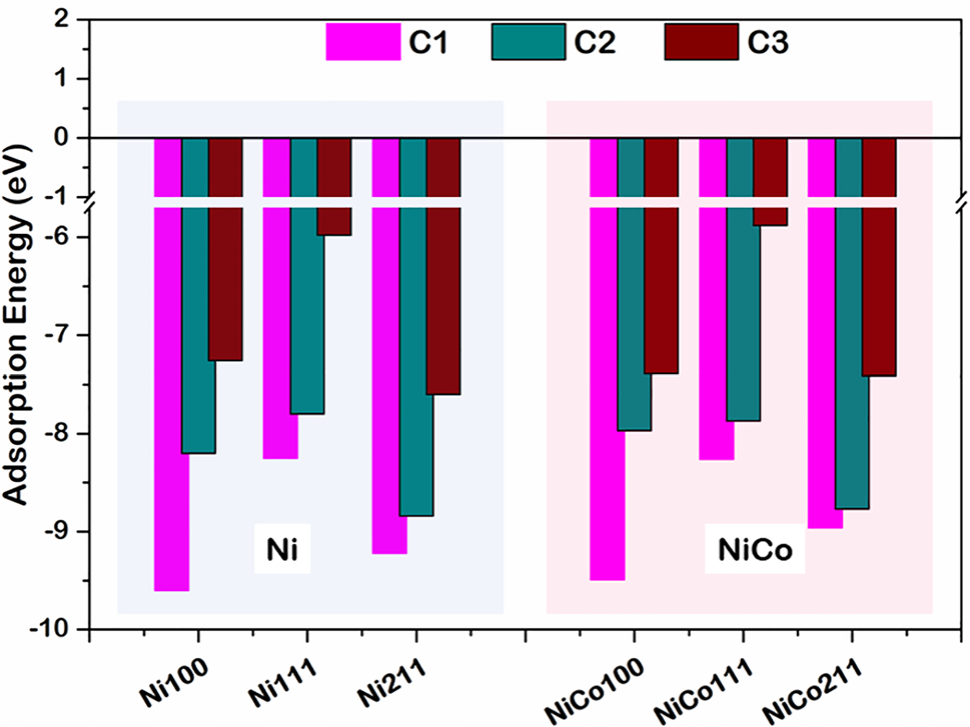
Adsorption energies (E_ads_) of C1, C2, and C3 coke species on Ni and NiCo surfaces.

The smallest coke classified by Bartholomew et al.^41,42^ is an alpha coke presented in the form of an atomic carbon element, which is the initial reactant to the formation of all types of coke. Regarding the first two steps of coke formation, the dimerization, which is the first condensation step of alpha carbon, will lead to the formation of the beta carbon or the amorphous carbon, where larger coke will form from such beta carbon. Thus, we modeled coke in forms of alpha and beta carbon as 1-atom (denoted as C1), 2-atom (denoted as C2), and 3-atom carbon (denoted as C3) adsorbed on the surface, where the binding strength of coke are determined denoted as E_ads_. The E_ads_ of various coke adsorbed on Ni and NiCo surfaces are compared, as illustrated in Fig. 4. Besides, E_ads_ for other active sites of the Ni and NiCo catalysts and the equilibrium adsorption height between C and the surface (d_c-sur_) were reported in Table S1 of the supplementary document. It was seen that both alpha and beta coke strongly chemisorbed with the E_ads_ ranging from − 1.0 to − 10.0 eV on all facets of both Ni and NiCo catalysts. Moreover, the adsorption strength decreases with the increasing size of coke from C1 to C3 on both catalysts. This is caused by an increased steric hindrance of larger coke when accumulated^43,44^. Among all facets, the (111) facet of both Ni and NiCo exhibited the weakest adsorption strength at all size of coke, suggesting that a weak bonding would form if the alpha coke accumulates on such surfaces and if the coke condensation proceeded to form a larger coke, the binding strength of such coke would still be weak suggesting the coke-resistant property of NiCo111 bimetallic catalyst to be comparable to that of the Ni111. Therefore, introducing Co forming NiCo helped resist the coke accumulation on the surface, which would be beneficial for a DRM process.

### Surface electronic properties of deactivated catalysts

#### Surface Bader charge and charge density difference analysis

**Figure 5:**
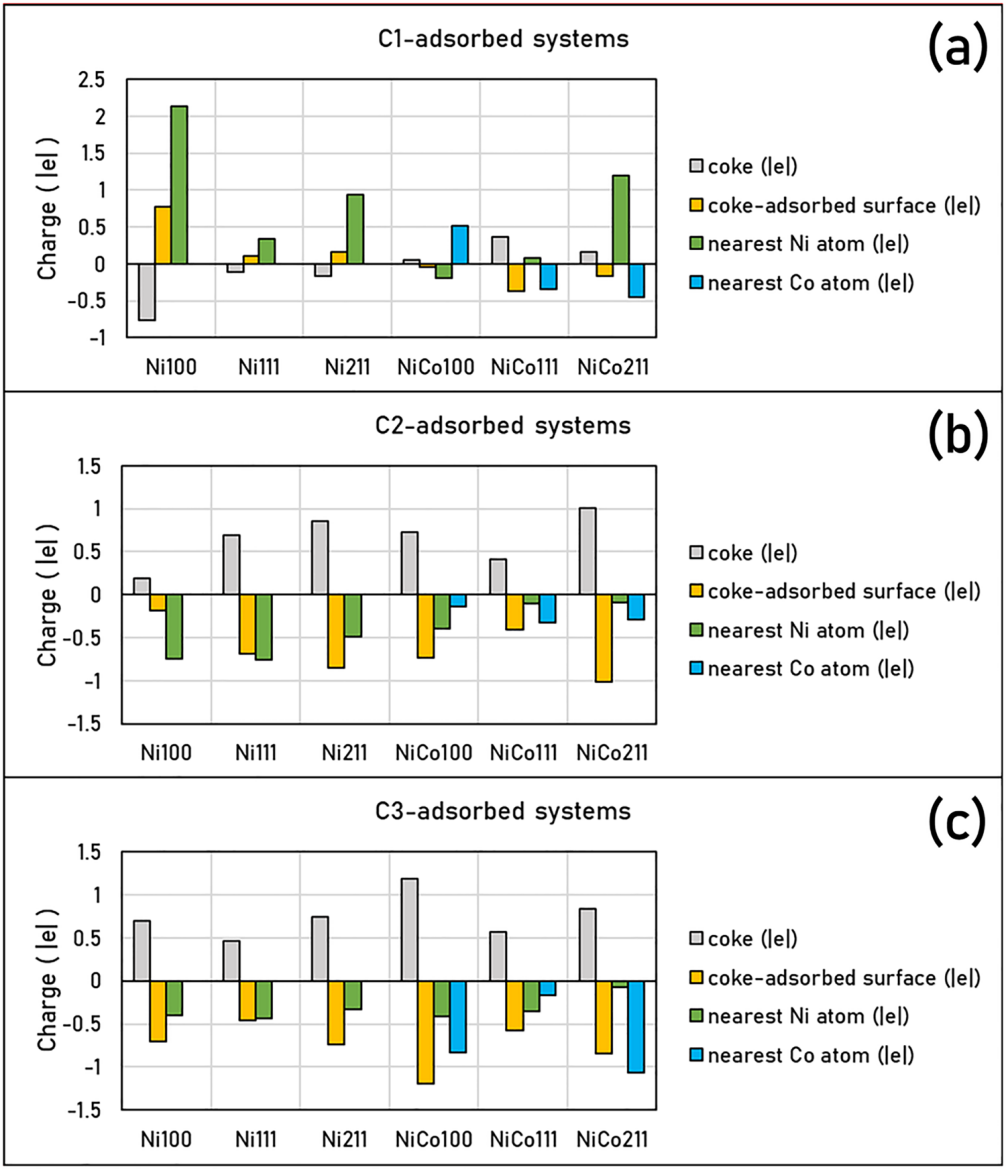
Bader charge analysis of cokes and coke-adsorbed Ni and NiCo surfaces, the nearest Ni atom (in the case of Ni and NiCo), and the nearest Co atom (in the case of NiCo): (**a**) C1-adsorbed surfaces, (**b**) C2-adsorbed surfaces, and (**c**) C3-adsorbed surfaces on Ni100, Ni111, Ni211, NiCo100, NiCo111, and NiCo211 surfaces.

**Figure 6:**
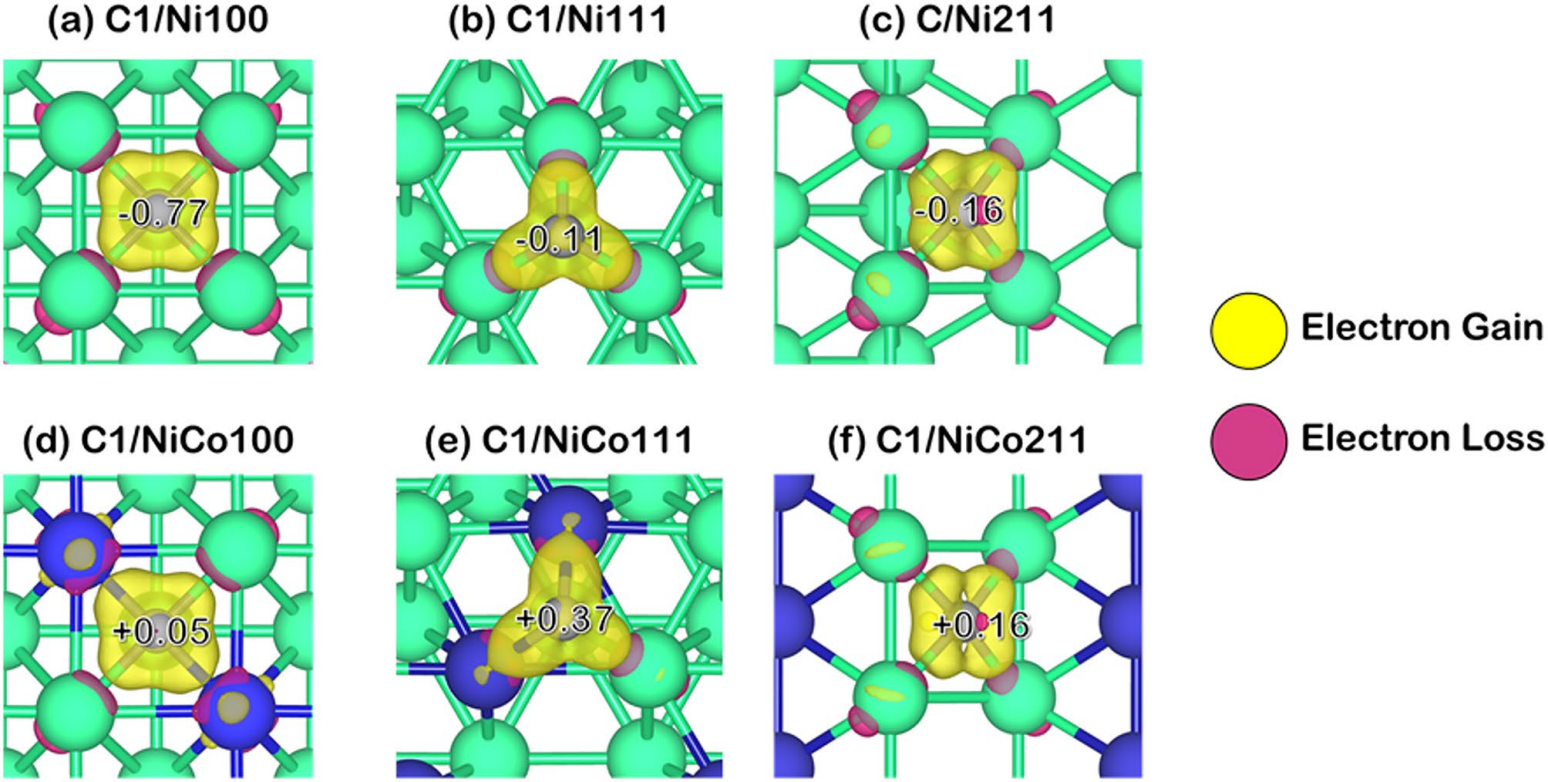
The charge density difference corresponding with Bader charge changes of C1 coke species adsorption on Ni and NiCo surfaces with an isovalue of ± 0.015 |e|/Å^3^.

**Figure 7:**
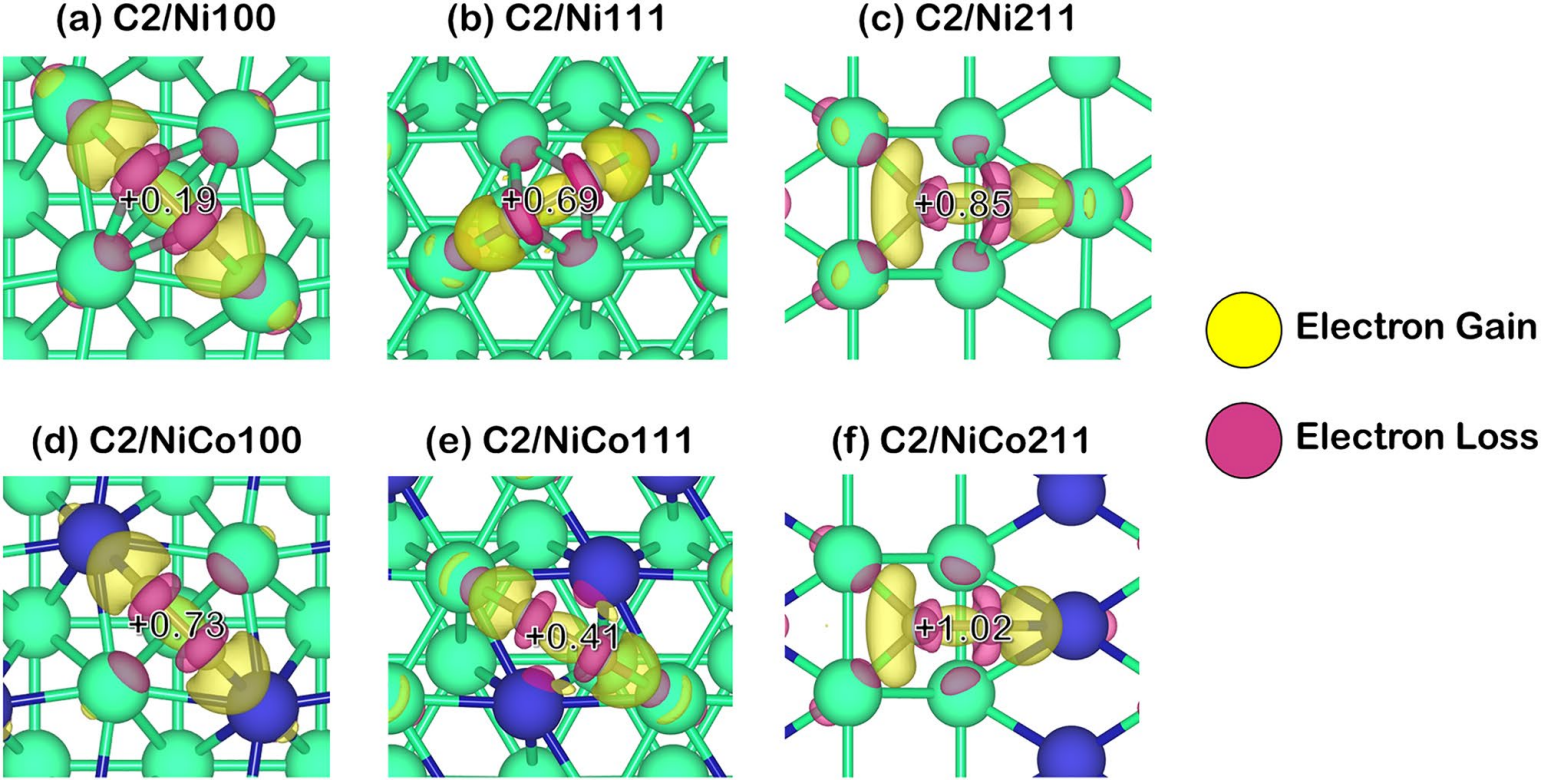
The charge density difference corresponding with Bader charge changes of C2 coke species adsorption on Ni and NiCo surfaces with an isovalue of ± 0.015 |e|/Å^3^.

**Figure 8:**
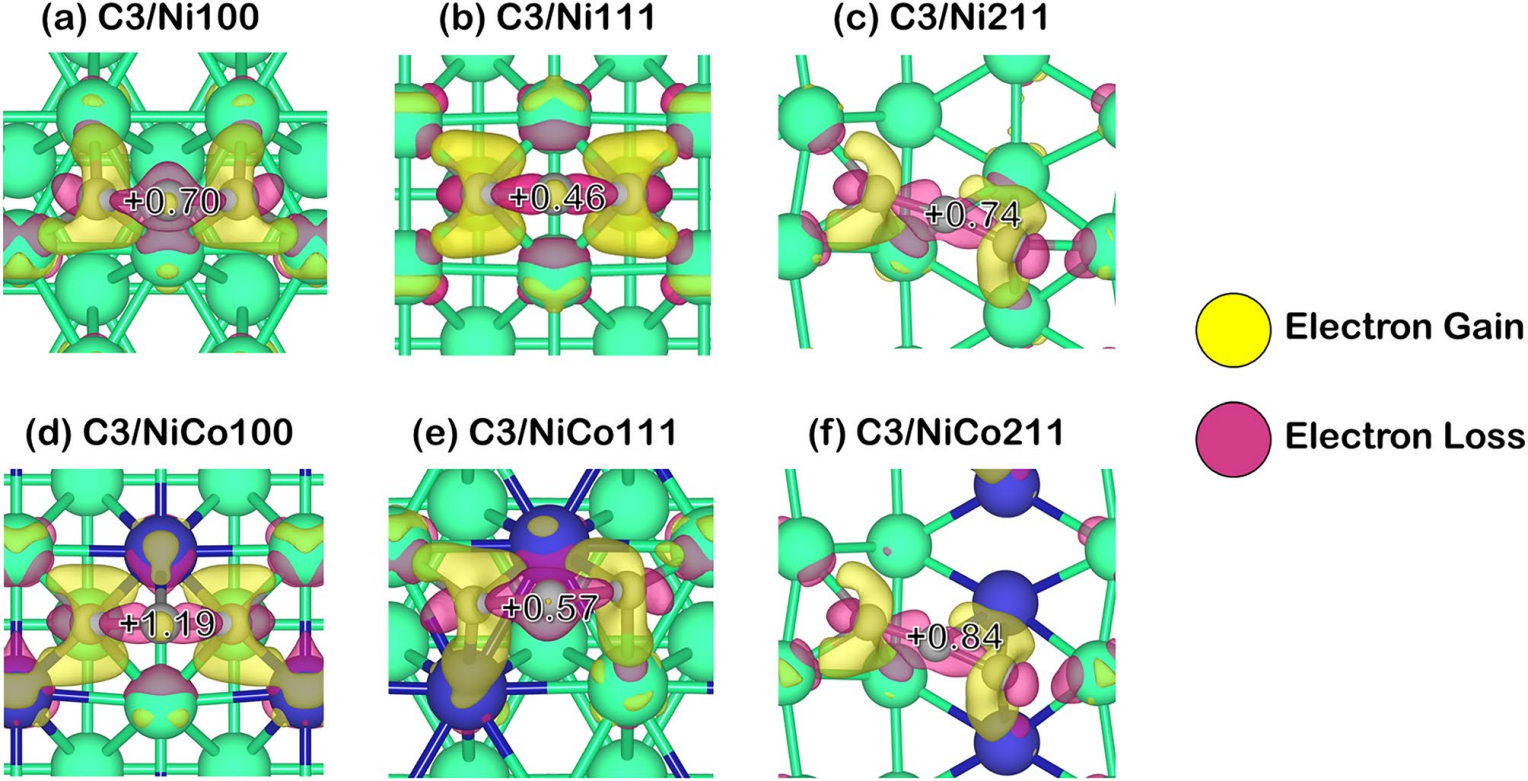
The charge density difference corresponding with Bader charge changes of C3 coke species adsorption on Ni and NiCo surfaces with an isovalue of ± 0.015 |e|/Å^3^.

Since the coke-resistant facets of Ni and NiCo catalysts providing weak coke adsorption have been determined, further understanding of how these cokes modified the active sites adsorbed must be obtained. Thus, we performed Bader charge analysis to investigate how the coke induces electrons on the catalyst surface as provided in Fig. 5, in which the Bader charge changes of each carbon species: C1, C2, and C3 were reported in Table 2. The additional information of Bader charges in these systems was summarized in Table S2 to Table S4 in the supplementary document, where positive values represented electron gain, while negative values represented electron loss as also described in computational details. Furthermore, charge density differences describing electron transfer during coke adsorption are exhibited in Figs. 6, 7 and 8. The electron gain and loss were represented as yellow and pink regions, respectively.

**Table 2:** The total charge of adsorbed 1-atom, 2-atom, and 3-atom coke species, and their individual C atom charge within the coke species all in the unit of electron.

Surface	Types of coke	Charges (|e|)			
		Total Coke	1st C atom	2nd C atom ^a^	3rd C atom ^a^
Ni100	C1	− 0.77	− 0.77	–	–
Ni111	C1	− 0.11	− 0.11	–	–
Ni211	C1	− 0.16	− 0.16	–	–
NiCo100	C1	0.05	0.05	–	–
NiCo111	C1	0.37	0.37	–	–
NiCo211	C1	0.16	0.16	–	–
Ni100	C2	0.19	0.42	− 0.24	–
Ni111	C2	0.69	0.19	0.49	–
Ni211	C2	0.85	0.48	0.36	–
NiCo100	C2	0.73	0.85	− 0.11	–
NiCo111	C2	0.41	0.21	0.20	–
NiCo211	C2	1.01	0.72	0.29	–
Ni100	C3	0.7	0.20	− 0.06	0.57
Ni111	C3	0.46	− 0.06	0.38	0.14
Ni211	C3	0.74	− 0.38	0.71	0.42
NiCo100	C3	1.19	0.39	0.06	0.74
NiCo111	C3	0.57	− 0.34	0.56	0.34
NiCo211	C3	0.84	− 0.48	0.42	0.90

^a^The charges of the 2nd C atom are shown for C2 and C3 since C1 represents atomic coke consisting of 1 atom, while the charge of the 3rd C atom is shown for the C3 system only.

From the charge density difference and Bader charge analysis on the adsorption of C1 on Ni and NiCo surfaces in Fig. 6, it was revealed that all Ni facets withdrew electrons from coke, resulting in a positive charge found on coke. Whereas the NiCo surfaces lost electrons to coke, yielding a negatively charged atomic coke. From this analysis, it can be inferred that the coke with a positive charge found in all Ni surfaces possessed the characteristics of sp^2^ carbon. This form of carbon would be less stable when compared to the negatively charged coke on NiCo, possessing an sp^3^ carbon form. Hence, the low stability of the coke on Ni surfaces would make the atomic coke more reactive to the reaction. Therefore, the condensation reaction forming larger cokes, which begins with the atomic coke adsorption^41,42^ would be more prone to proceed on the Ni surface than that on the NiCo surface due to the lower stability sp^2^ carbon of the adsorbed atomic coke on the Ni, and this may explain why carbon deposition is high on Ni compared to the NiCo system.

Moreover, the analysis of electron transfer on the 2-atom (C2) and 3-atom (C3) coke adsorption showed a similar trend as described in Figs. 7 and 8. These cokes gained electrons, becoming a negatively charged carbon while withdrawing nearby electrons from Ni active sites on other regions of the surface and Ni and Co active sites in the NiCo surface. Apart from the charge analysis on the overall coke molecule, the electron transfer on each carbon atom in such a molecule showed that almost all carbon atoms in the coke molecule gained electrons resulting in the majority of the carbon atom being in the sp^3^ form. Thus, the charge transfer in atomic and higher coke adsorbing on the Ni and NiCo system suggested high coke-resistant property of NiCo surface evident from a more stable coke in a positively charged form leading to a more stable sp^3^ form of carbon compared to the sp^2^ form in the Ni surface.

#### Surface projected density of states (PDOS) analysis

**Figure 9:**
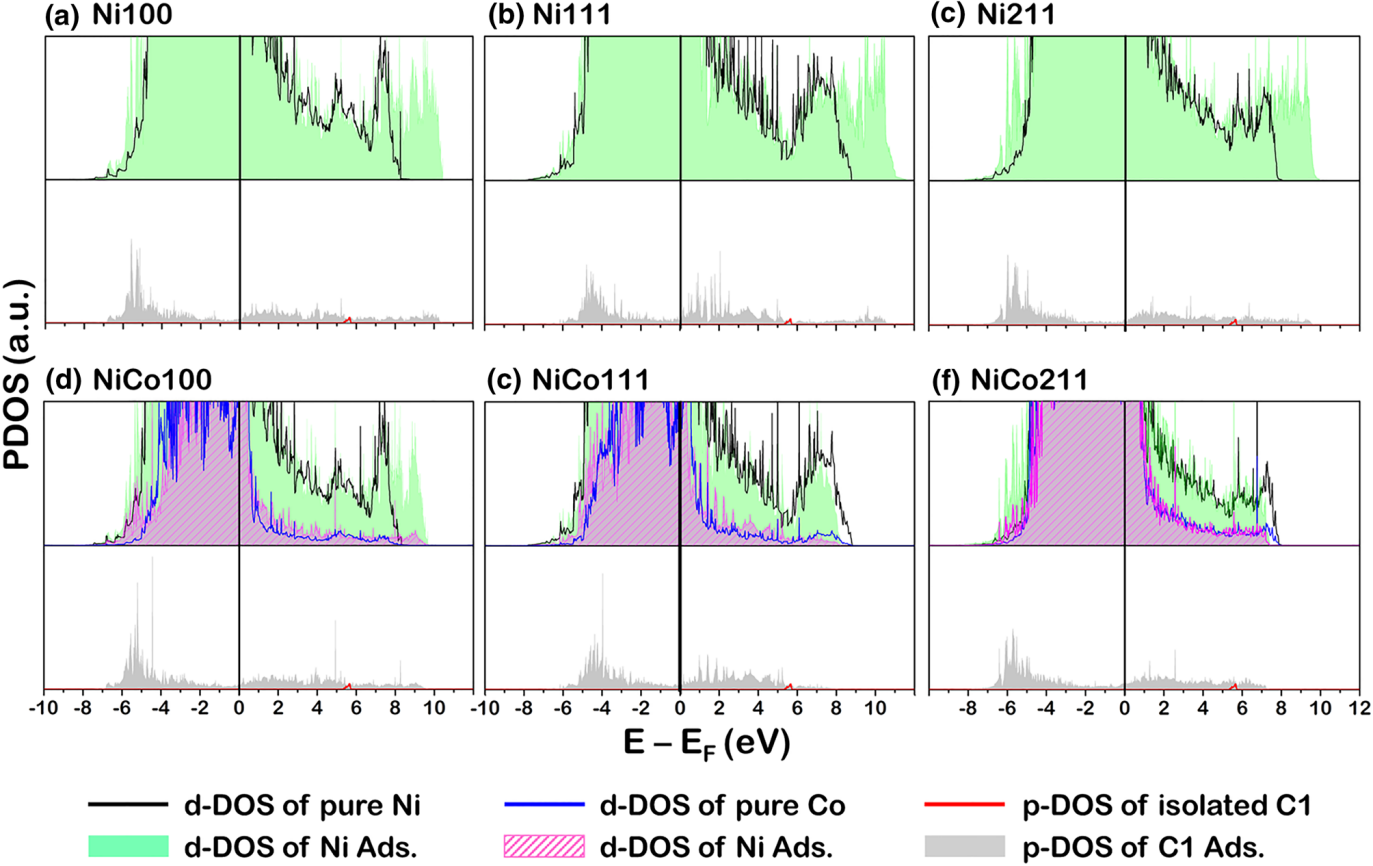
The PDOS profiles of fresh and 1-atom-carbon (C1)—adsorbed catalyst surface: (**a**) Ni100, (**b**) Ni111, (**c**) Ni211, (**d**) NiCo100, (**e**) NiCo111, and (**f**) NiCo211 surfaces. The top panel is the PDOS of the fresh and coked surface, while the bottom panel is the isolated and adsorbed C1 atom.

**Figure 10:**
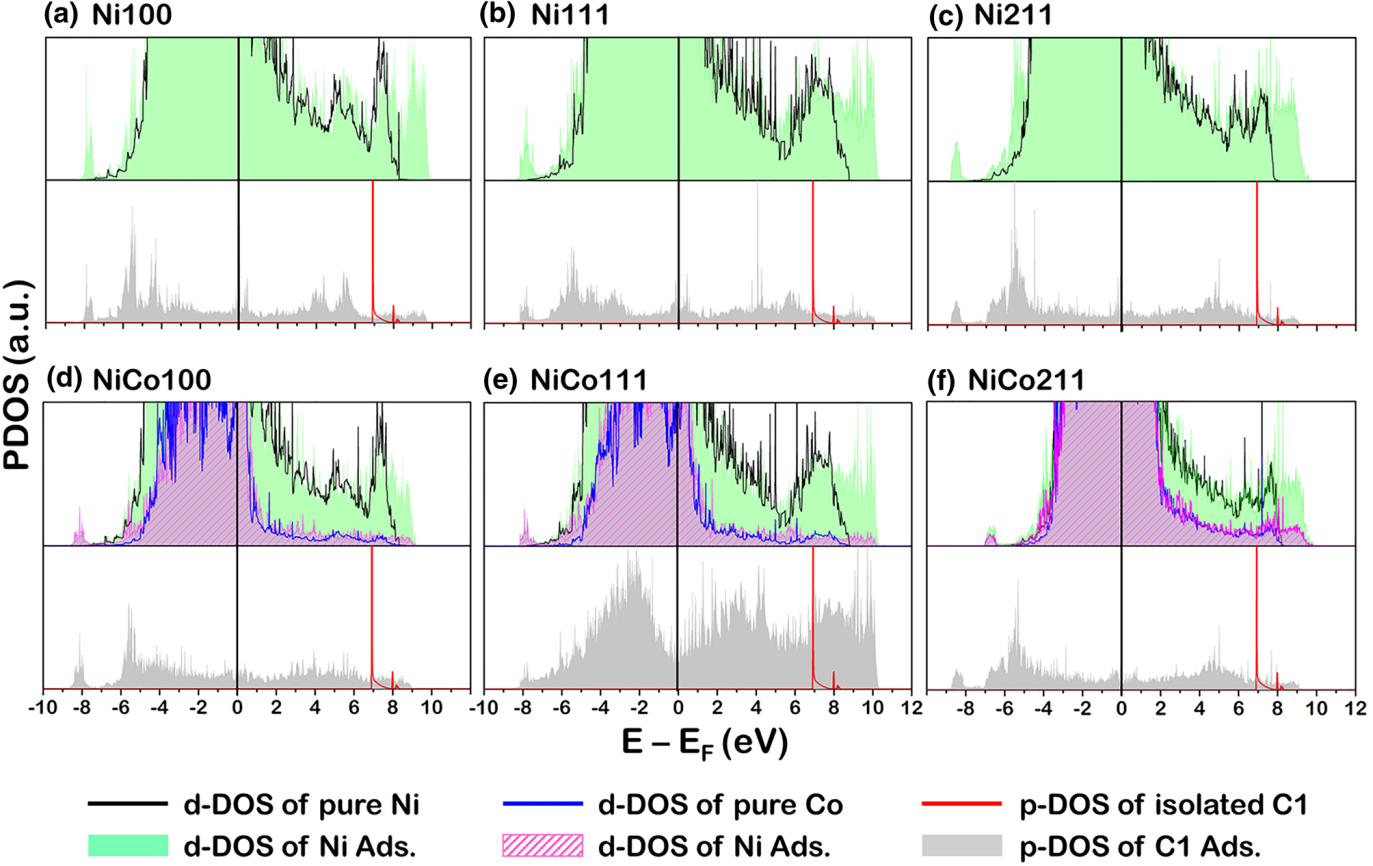
The PDOS profiles of fresh and 2-atom-carbon (C2)—adsorbed catalyst surface: (**a**) Ni100, (**b**) Ni111, (**c**) Ni211, (**d**) NiCo100, (**e**) NiCo111, and (**f**) NiCo211 surfaces. The top panel is the PDOS of the fresh and coked surface, while the bottom panel is the isolated and adsorbed C2 molecule.

**Figure 11:**
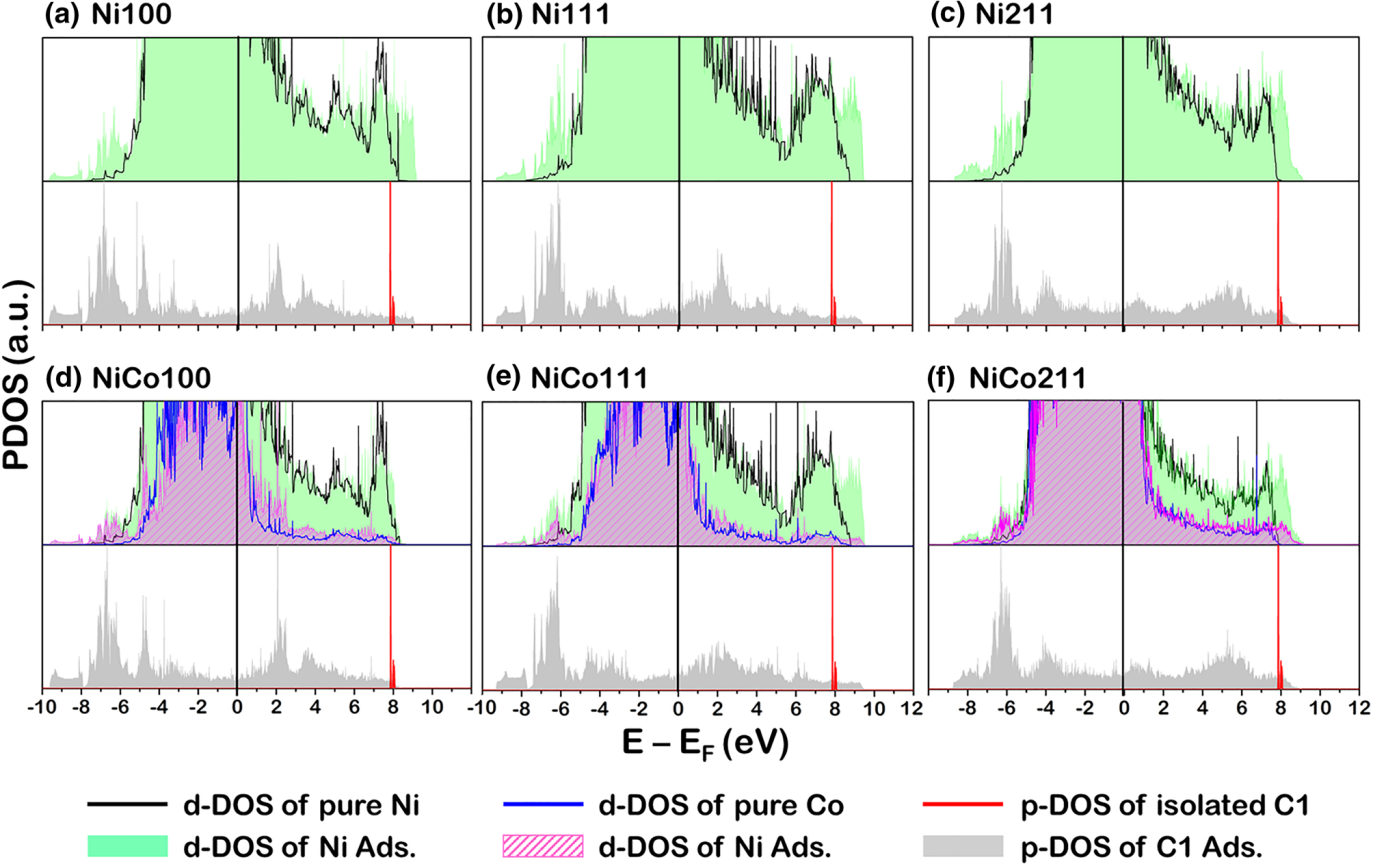
The PDOS profiles of fresh and 3-atom-carbon (C3)—adsorbed catalyst surface: (**a**) Ni100, (**b**) Ni111, (**c**) Ni211, (**d**) NiCo100, (**e**) NiCo111, and (**f**) NiCo211 surfaces. The top panel is the PDOS of the fresh and coked surface, while the bottom panel is the isolated and adsorbed C3 molecule.

Apart from the information on the electron transfer in the system when coke is present, the interaction between the C atom and the catalyst surfaces is investigated through PDOS profiles, as illustrated in Figs. 9, 10 and 11. In each profile, the valence state of each element, viz the p-orbital projected density of states (p-PDOS) of coke (carbon atom) and the d-orbital projected density of states (d-PDOS) of Ni and Co were presented. Note that the PDOS of each type of element in Figs. 9, 10, and 11 is the sum of individual d-PDOS in the case of Ni and Co element and individual p-PDOS in C element. The following notation was used to illustrate the PDOS profiles of fresh and coke-adsorbed Ni and NiCo surface. The Ni d-PDOS in fresh Ni and NiCo surfaces shown in Figs. 9a–f, 10a–f, and 11a–f, the d-PDOS profiles were illustrated as a solid black line, while the Co d-PDOS of fresh NiCo surfaces in Figs. 9d–f, 10d–f, and 11d–f, they are plotted as a solid blue line. The d-PDOS of Ni d-PDOS in coke-adsorbed Ni and NiCo is plotted as a green space-filling shape, while the d-PDOS of Co d-PDOS in coke-adsorbed NiCo are plotted as a purple space-filling shape.For the p-PDOS profiles of coke, the isolated coke atom (in the case of C1) and molecule (in the case of C2 and C3) were plotted as a solid red line, while the adsorbing coke is plotted as the gray space-filling shape. Based on such notation of PDOS profiles, the analysis of the fresh and coked Ni100, Ni111, and Ni211 suggested the interaction between all sizes of coke and the Ni surface atoms determined via the overlapping Ni d-PDOS (green space-filling) and C p-PDOS (gray space-filling). For instance, in the case of atomic coke adsorption, the major overlapping PDOS is located around − 7.0 to − 4.0 eV for the Ni100 and Ni211 facets shown in Fig. 9a,c, while Ni111 showed the major overlapping PDOS at − 6.0 to − 3.0 eV. For the 2-atom coke adsorption, the major overlapping peaks around − 8.5 eV for Ni211 and -8.0 for Ni100 and Ni111 were detected, whereas the 3-atom coke adsorption showed major overlapping peaks around − 8.0 and − 6.0 eV for all Ni facets. Thus, from the overlapping in all Ni surfaces, the bonding between the Ni atom on the Ni surfaces and the adsorbed C atom was confirmed.

On the interaction between coke and the NiCo catalyst surface, the Ni d-PDOS, Co d-PDOS, and C p-PDOS profiles in Figs. 9d–f, 10d–f, and 11d–f were analyzed. Similar to the PDOS in the case of Ni catalyst surfaces, the overlapping between coke and both metal active sites: Ni and Co was observed. For example, the atomic coke (C1) adsorption on the NiCo surface showed an overlapping PDOS peak of C p-PDOS to both Ni d-PDOS and Co d-PDOS at around − 5.0 eV for NiCo100, NiCo111, and NiCo211 facets. Hence, the partial overlapping in the NiCo of C p-PDOS with Ni d-PDOS and Co d-PDOS indicated Ni-C and Co–C bonding, in which the adsorption of C2 in Fig. 10 and C3 in Fig. 11 indicated the same conclusion. Interestingly, compared to the Ni-C bonding in the Ni monometallic surface (subfigure (a–c) of Figs. 9, 10, and 11), the height of such overlapped Ni-C peak in the NiCo system is observed to be lower. (subfigure (d–f) of Figs. 9, 10, and 11). Besides, these PDOS profiles together with a weak E_ads_ of coke found in all NiCo surfaces suggested a promotional role of Co which is to weaken the interactions between the Ni and C atoms, which it would be beneficial for the Ni active site since the site blockage is less likely to occur, increasing available sites for the main reaction.

### Coke mobility on Ni and NiCo surfaces

While the carbon adsorption strength provided the information of the most stable adsorption site, the energy needed for C atom diffusion is also an essential parameter to understand the propagation of coking as well as how easily the coke can be removed from the catalyst since it is an initial step towards coke formation^41,42^. Therefore, the mechanism of C diffusion on Ni and NiCo surfaces was discussed in this section through the coke mobility scheme defined in terms of the activation energy (E_a_) of carbon moving from the most stable adsorption site, the strongest C adsorption site to the possible nearby active site. The activation energies of forward (E_a,f_) and reverse (E_a,r_) coke diffusion, as well as the heat of the diffusion process (∆H), are provided in Table 3. Because the diffusion of adsorbed C atom started from the most stable adsorption site, diffusion to other possible adsorption sites are all endothermic. Besides, all possible diffusion pathways, the E_a,f_, E_a,r_, and imaginary frequency, are summarized in Table S5.

**Table 3:** The activation energies (E_a_) of the forward (E_a,f_) and reverse (E_a,r_) reaction for C movement and heat of coke diffusion (∆H) on the Ni111, Ni211, Co001, NiCo111, and NiCo211 surfaces.

Catalysts	Surface	Elementary step	E_a,f_ (eV)	E_a,r_ (eV)	∆H(eV)
Ni	111	C_HCP_ → C_FCC_	0.50	0.39	0.11
	211	C_4-fold_ → C_3-fold_	1.42	0.34	1.09
Co	001	C_HCP_ → C_3-fold_	0.42	0.19	0.23
Ni-Co	111	C_HCPNi1_ → C_FCCNi1_	0.34	0.19	0.16
	211	C_4-fold_ → C_3-foldNi2_	1.01	0.26	0.74

Considering all clean Ni facets of the Ni monometallic catalyst, it was found that the C atom prefers to adsorb on the fourfold of Ni100, HCP of Ni111, and fourfold site of Ni211. However, on the Ni100 surface, the movement of C starting from the fourfold site is not considered since such a surface only has one stable adsorption site. For other facets, the C atom on the Ni111 can move from HCP to FCC site with an E_a,f_ of 0.50 eV, while the C diffusion from a fourfold to a threefold hollow site on the Ni211 surface required an E_a,f_ of 1.42 eV, indicating that the coke mobility on the flat Ni111 surface is easier than that of the stepped Ni211 surface.

Such easy C diffusion on the flat surface is similar in the case of NiCo surface, in which the E_a,f_ of C atom on NiCo111 and NiCo211 is 0.34 and 1.01 eV, respectively. When comparing the E_a,f_ of analogous surfaces: Ni111 and NiCo111, it was found that the presence of Co in the NiCo surfaces lowered the E_a,f_ of C diffusion, indicating an increase in coke mobility. This enhanced coke mobility on NiCo surfaces is considered beneficial since easy diffusion of coke on the surface would increase the chance of coke encountering the coke-removing species, i.e., H or O atom led to hydrogenation and oxidation of coke.

**Figure 12:**
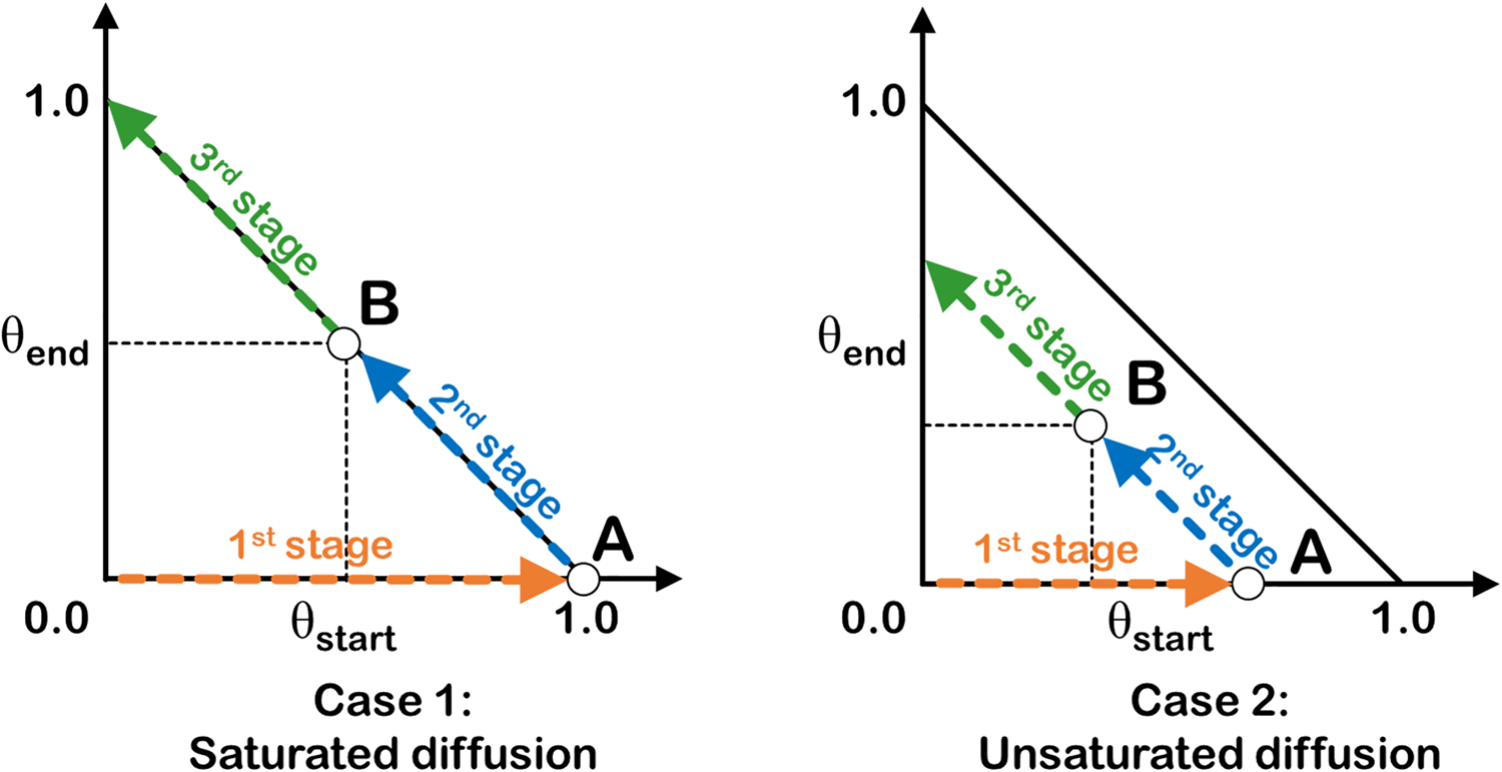
Coke mobility scheme (CMS) illustrated by the surface coverage of C at the initial and terminal diffusion sites, where two cases: (1) saturated diffusion and (2) unsaturated diffusion, were proposed for surface C diffusion in all system. (The 45-degree solid line marked the limit for the maximum total surface coverage of C of such two sites that is equal to unity).

The coke mobility scheme (CMS) in Fig. 12 is proposed to describe coke diffusion on each surface, where three stages of coke diffusion on the surface are considered. Besides, two diffusion conditions: the saturated and unsaturated diffusion of coke are considered based on the maximum accumulation of coke at the initial diffusion site reflected through the parameter θ_C,start-max_ that ranges from zero to one, where the saturated diffusion and unsaturated diffusion started with the θ_C,start-max_ value of one and less than one, respectively. The three-stage coke diffusion comprised the coke accumulates at an initial diffusion site as a 1st stage, in which the most stable C adsorption site is designated to be the initial site. During this stage, the coke built up, and the C surface coverage increased at the initial diffusion site (θ_C_,_start_) up to its maximum theoretical capacity of θ_C,start-max_, while in this stage, the diffusion from this initial site to the terminal sites had not occurred yet. Thus, the surface coverage of C at the terminal diffusion site (θ_C_,_end_) is zero. Propagating into the 2nd stage, coke at an initial adsorption site started to diffuse to the terminal diffusion site. At this stage, the amount of coke on both initial and terminal sites determines the driving force of the forward and reverse diffusion before the diffusion ceased when these rates are equal. Ultimately, when there is high enough coke at the terminal site, the coke will diffuse back to an initial site as the reverse diffusion rate is larger than the forward one. This marked the starting point of the 3rd stage.

Another possible condition of CMS is the unsaturated coke diffusion shown on the right panel of Fig. 12. This case is the special case of saturated diffusion. The coke diffusion from the initial to the terminal sites occurred before the accumulation of the coke at the initial site reaches its maximum of one. Apart from this, the 2nd and 3rd stages can be described via the saturated diffusion case. However, as the diffusion model separated the 2nd and 3rd stage at equal θ_C_,_start_ and θ_C_,_end_ of $\frac{{1}}{{2}}$(θ_C,start-max_), this description is only valid for the surface of diffusion with the same value of the forward and reverse rate constant; in other words, the identical activation energy of forward and reverse C diffusion. Therefore, the rate of diffusion of Ni, Co, and NiCo must be determined to describe the 3-stage C diffusion on each surface and to indicate the surface that facilitates such diffusion, enhancing the coke-resistance. Moreover, on each surface, the 2nd stage ended when the rate of the C diffusion from the initial site becomes negative.

**Figure 13:**
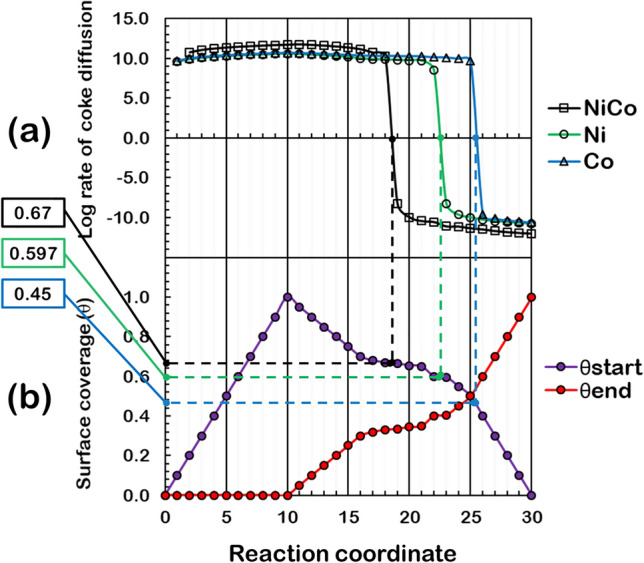
Coke mobility of Ni and NiCo catalysts represented by (**a**) the log rate of coke diffusion from the most stable C adsorption site to the nearby site for all catalysts and (**b**) the surface coverage of carbon atom of the initial and terminal diffusion site during 1st, 2nd, and 3rd stage of coke surface diffusion.

Thus, we can determine how fast the back-diffusion occur on a surface by reading the x-axis in the plot (a) of Fig. 13 when the rate became zero and use this value of x on the plot (b) of Fig. 13 to determine the maximum surface coverage at the terminal site before the back-diffusion occurred. According to this, the surface with the highest surface coverage of the terminal site before back-diffusion would prefer since this situation would avoid having coke at the initial site of strong coke binding, preventing poison of the site from coke.

The calculated rate of coke diffusion on the pure surface from Eq. (7) was obtained in each stage for Ni, Co, and NiCo surfaces and plotted parallel to the CMS show in Fig. 13. It was revealed that in the 1st stage of coke diffusion, the NiCo system exhibited the highest rate of diffusion, followed by Ni and Co systems, which are comparable. For all systems, the maximum rate of forwarding coke diffusion is at the same reaction coordinate when the C surface coverage of the initial site is equal to unity, which marked the end of the 1st stage. During the 2nd stage of coke diffusion, the diffusion rate began to slow down for all systems due to an increased θ_C,end_ reflecting the coke accumulation at the terminal site. Besides, at a specific point of the 2nd stage, the rate of the reverse diffusion exceeded the forward rate causing the movement of the carbon atom back to an initial site. Among these three surfaces, the back-diffusion began first on the NiCo surface at θ_start_ of 0.67, followed by Ni at θ_start_ of 0.597 (solid green line), and Co at θ_start_ of 0.45 (solid blue line), respectively, where this marked the end of the 2nd stage and the beginning of the 3rd stage. In the last stage, the NiCo surface exhibited the highest rate of back-diffusion, followed by Ni and Co surfaces. This can be implied that the NiCo surface can promote the forward coke diffusion as much as the reverse one suggesting high coke mobility on this system.

**Figure 14:**
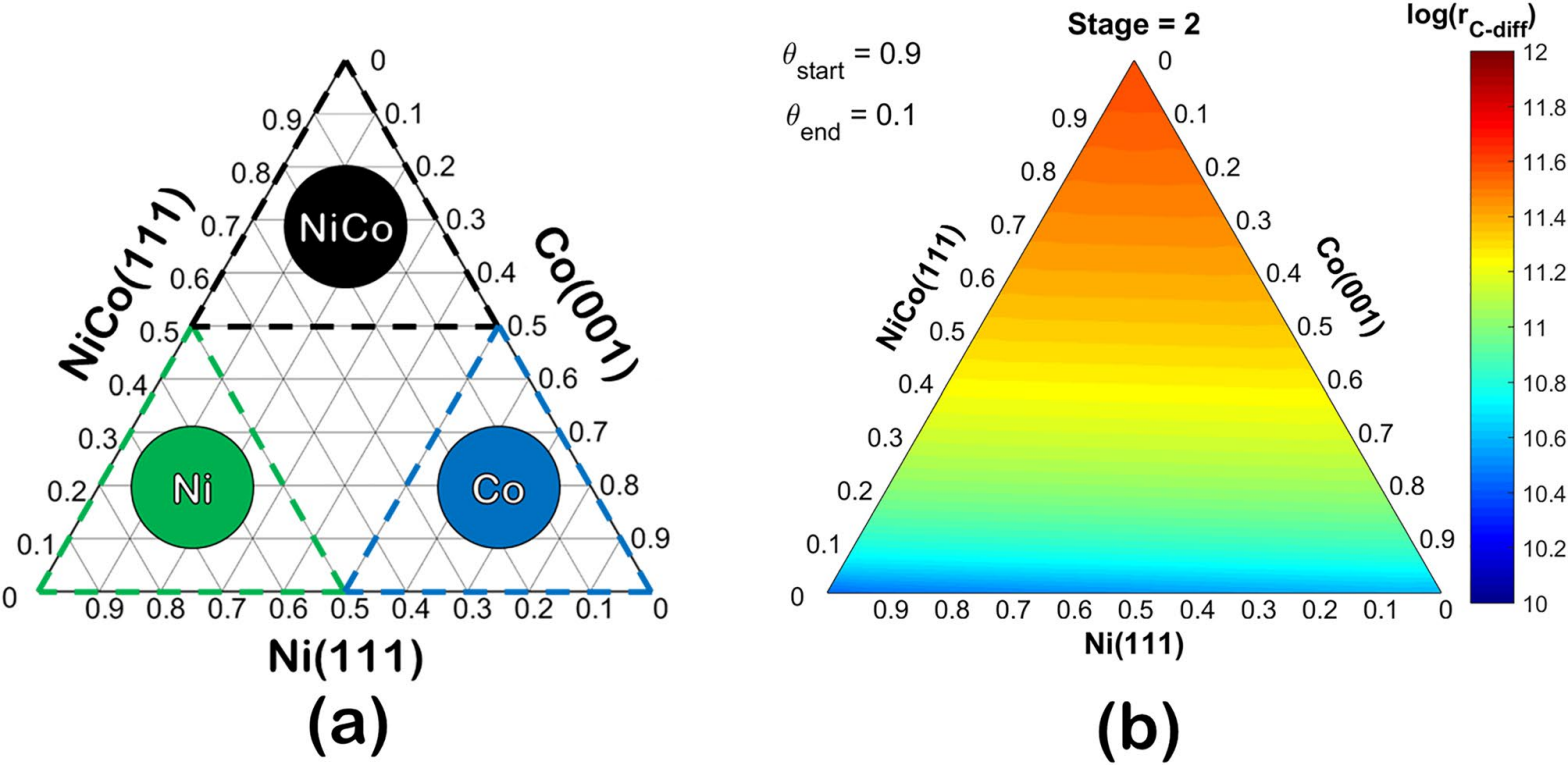
Ternary contour plot (TCP) exhibiting (**a**) three zones of dominant 111-analogous surface in the NiCo bimetallic catalyst: Ni111-dominant, Co111-dominant, and NiCo111-dominant, and (**b**) the rate of surface carbon diffusion (log(r_C-diff_)) as a function of the Ni111:Co111:NiCo111 surface fraction at a specified initial site surface carbon coverage (θ_start_), terminal site surface carbon coverage (θ_end_), where the low to high log rate is from the color of blue to red in the colored bar.

**Figure 15:**
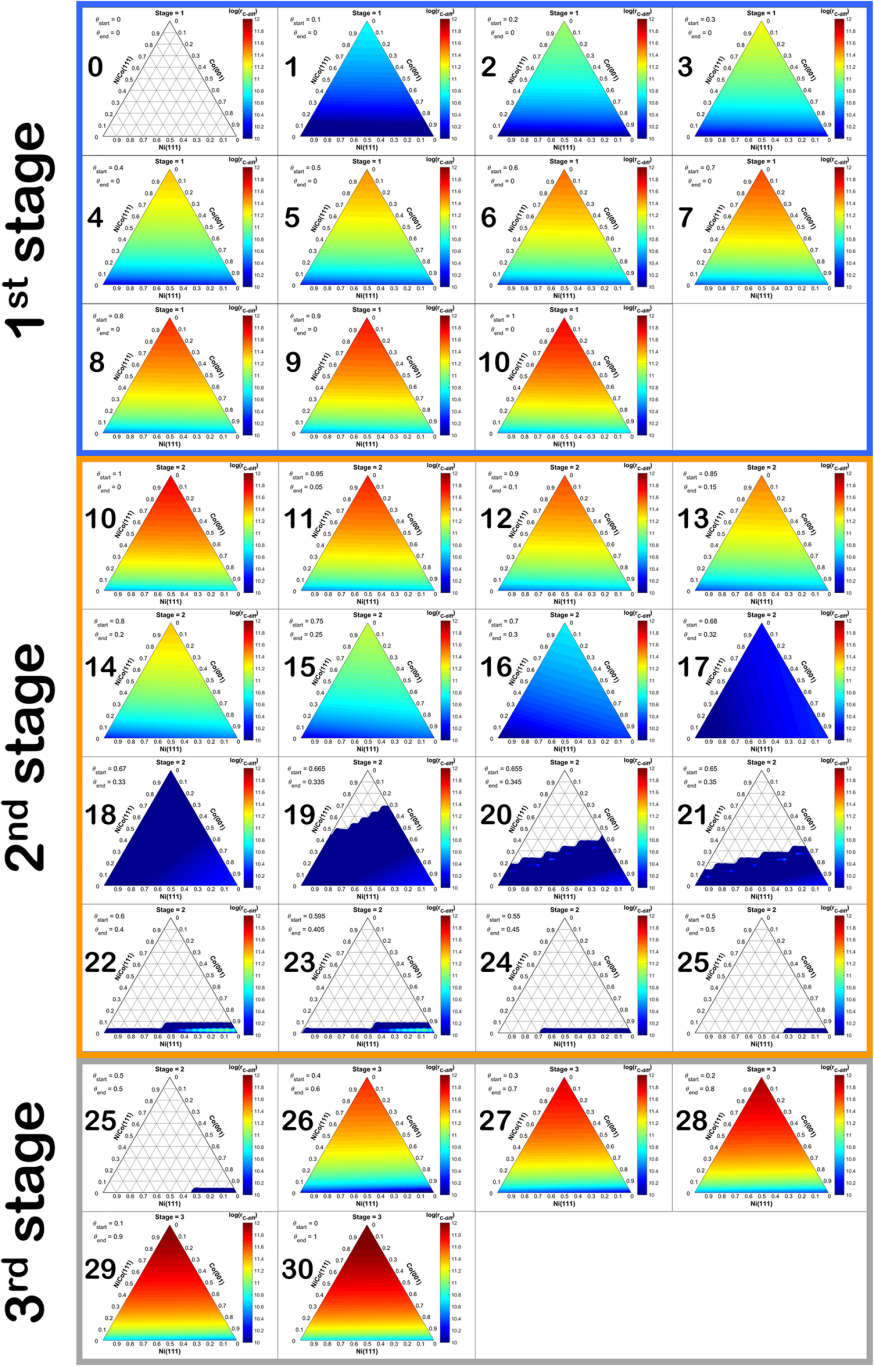
Ternary contour plot (TCP) of log rate of C diffusion as a function of the surface composition comprises of Ni111, Co111, and NiCo111 during the 1st (frame 0–10), 2nd (frame 10–25), and 3rd (frame 25–30) stage carbon diffusion, where each TCP corresponds to the reaction coordinate in Fig. 13. Note that the rate of diffusion in the 3rd stage is the rate of the back-diffusion; thus, the direction of diffusion is from the terminal site to the initial site. Besides, all TCP are shown individually in the Supplementary document in Figs. S2–34.

The NiCo bimetallic catalyst prepared by the method described in the experimental details would result in not only the NiCo region but also comprised the Ni and Co monometallic regions left from the synthesis. Therefore, we describe the catalyst surface via four main regions exhibited as a ternary plot and 3-dimensional ternary contour plot (TCP) shown in Fig. 14a,b, respectively. The ternary plot in Fig. 14a presented four main regions: (1) Ni-dominant, (2) Co-dominant, (3) NiCo-dominant, and (4) the mixed region situated in the middle of the ternary plot. Regarding these regions, the rate of surface coke diffusion is calculated from Eq. (8) to describe the three-stages of coke diffusion in each Ni, Co, or NiCo-dominant region as illustrated in Fig. 15, in which the number in each TCP corresponded to each reaction coordinate along three stages of diffusion in Fig. 13.

It was found that during the 1st stage, the region with NiCo-dominant facilitates the diffusion of coke the most reflected from the highest rate of diffusion, while the diffusion rate in Ni-dominant is comparable to the Co-dominant. In the 2nd stage, as the coke at the terminal site started to accumulate, the rate of coke diffusion is reduced for all regions. Moreover, the back-diffusion began first in the NiCo-dominant region as the negative rate is observed, followed by the Ni-dominant, and Co-dominant region, respectively. This situation correlated with the carbon diffusion in the pure surface described in Fig. 13. In the last stage, all TCP described the rate of back-diffusion; hence, the direction of carbon diffusion is from the terminal site to the initial site. It can be observed that the rate of the back-diffusion is highest in the region of NiCo-dominant, followed by the Ni-dominant and Co-dominant, respectively. Thus, to enhance the stability against the coking of the surface, the synthesis method should yield the catalyst surface with the majority of the surface being NiCo-dominant while minimizing the Ni and Co monometallic regions.

In summary, the high coke resistance found from the experiment on the NiCo system can be explained through its fast carbon diffusion together with its weak coke adsorption strength. Nevertheless, the removal of coke via oxidation and hydrogenation combined with fast coke diffusion on the surface would lower the chance of an active site being blocked by coke species, enhancing the catalyst’s stability.

## Conclusion

In this work, the coke-resistant property of the NiCo catalyst was investigated via experiments and density functional theory based calculation. The strength of coke adsorption was reduced on the NiCo for all sizes of coke: atomic coke, 2-atom, and 3-atom coke species. Hence, the Co active site in NiCo help to weaken the bonding between an adsorbed coke species to the Ni active site, in which in the case of pure Ni catalyst, it is observed to be very strong. Interestingly, another role of Co is to facilitate the coke movement on the catalyst surface, reducing the chance of coke deposition on Ni active site comparing to the pure Ni and Co surfaces during the DRM process. However, as the NiCo surface exhibited both high diffusion and back-diffusion rate than that of the Ni and Co surfaces, it suggested that the NiCo needed a lower critical amount of coke at the terminal diffusion site to initiate the back-diffusion than the Ni surfaces and the removal of the coke at the terminal site should be managed. For the analysis of carbon diffusion based on a real particle, four regions of the NiCo particle are proposed: (1) Ni-dominant, (2) Co-dominant, (3) NiCo-dominant, and (4) the mixed regions comprised comparable amount of Ni, Co and NiCo surfaces. It was found that the presence of a NiCo-dominant region should be achieved when one synthesized the NiCo catalyst since it played an essential role in facilitating the coke diffusion. The Ni-dominant and Co-dominant regions provided a very low coke diffusion and should be kept low in amount when preparing the catalyst. Therefore, this work provided the key understanding behind the high stability of NiCo system that the coke diffusion is facilitated, increasing the availability of the Ni active site for the main reaction. Removing coke via either oxidation or hydrogenation should always be combined with this high coke mobility to prevent a fast back-diffusion on the NiCo surface. Future works deal with the optimal surface composition of this NiCo system that would both facilitate the coke diffusion and coke removal via hydrogenation and oxidation.

## Methodology

### Experimental details

#### Catalyst preparation

The γ-Al_2_O_3_-HY-zeolite supported catalyst was prepared by a sol–gel method using aluminium isopropoxide (Aldrich) and ethanol (Merck) as a solvent. The HY (faujasite) zeolite with a Si/Al atomic ratio of 100 (TOSHO) was used as-is. The Ni, Co, and NiCo catalysts were prepared by incipient wetness impregnation using Ni(NO_3_)_2_·6H_2_O (Aldrich) and Co(NO_3_)_2_·6H_2_O (Aldrich) as Ni and Co precursors, respectively. The Ni:Co metal loading was varied at 100:0, 30:70, 50:50, and 0:100. After impregnation, catalysts were dried at 110 °C for 24 h prior to the calcination under airflow at 550 °C for 2 h.

#### Catalyst characterization

A JEOL JSM-5800LV scanning electron microscope with energy dispersive X-ray analysis (SEM–EDX) was used to probe the catalysts’ morphology and the elemental distribution. The sample was subjected to thermogravimetric analysis (Diamond Thermogravimetric and Differential Analyzer) and to determine carbon deposition (heating from 30 °C to 1000 °C at a rate of 10 °C/min in a 100 ml/min in synthetic air).

#### Catalysis performance of Ni and NiCo for DRM reaction

The DRM reaction was performed using a continuous-flow quartz reactor, where the catalyst was packed on quartz wool equipped in the middle zone of the quartz tube reactor. Then, the packed catalyst was reduced at 500 °C for 1 h. with a continuous flow of H_2_ at the constant flow rate of 50 ml/min. The reaction was started by removing remained H_2_ using 50 ml/min of N_2_ before heating the reactor to 700 °C. Afterward, the 1:1 volume ratio of CH_4_:CO_2_ was continuously injected into the reactor with a total flow rate of 60 ml/min. The reaction was carried out under atmospheric pressure for 3 h. When the reaction was completed, the compositions of remained precursors and all products were analyzed via Thermal Conductivity Detector (TCD) type gas chromatograph (Shimadzu, GC-8A) equipped with Porapak-Q and Molecular sieve 5A packed column, in which the Argon was employed as a carrier gas.

### Computational details

#### Density functional theory-based calculations

Spin-polarized DFT was performed using the Vienna ab initio simulation package (VASP)^45–47^ based on the projector augmented wave (PAW). The exchange–correlation functional with generalized gradient approximation (GGA) by Perdew, Burke, and Ernzerhof (PBE) was used. The main parameters used in this work include cut-off energy of 400 eV, an energy convergence of 1.0 × 10^–6^ eV, and 3 × 3 × 1 Monkhorst–Pack *k*-mesh Brillouin-zone integration^48^. All structures were optimized using the conjugate gradient method^49^, in which all geometries were relaxed until the force convergence was less than 0.01 eV/Å. Van der Waals dispersion correction was applied using the DFT-D3 method proposed by Grimme et al.^50^. The interaction between coke and catalyst surface was determined by the adsorption energy (E_ads_) of one, two, and three carbon atoms on the Ni100, Ni111, Ni211, Co001, NiCo100, NiCo111, and NiCo211 surfaces, calculated as follows:

2 $${\text{E}}_{{{\text{ads}}}} = {\text{E}}_{{{\text{complex}}}} - ({\text{E}}_{{{\text{coke}}}} + {\text{E}}_{{{\text{surface}}}} )$$

The parameter ${\text{E}}_{{{\text{complex}}}}$ is the total energy of the C adsorbed surface, ${\text{E}}_{{{\text{coke}}}}$ denotes the total energy of an isolated coke atom/molecule in a vacuum with the cell size of 30 × 30 × 30 Å^3^, and ${\text{E}}_{{{\text{surface}}}}$ is the total energy of the clean catalyst surfaces before adsorption. A negative value of ${\text{E}}_{{{\text{ads}}}}$ indicates the favorable adsorption, while the positive value suggested desorption of the adsorbate. To determine the surface species that acted as electron donors and acceptors during coke adsorption on the surfaces, the partial charge between C atom adsorbed surface ($\Delta \updelta_{{\text{C}}}$) and catalyst surfaces ($\Delta \updelta_{{{\text{Sur}}}}$) was calculated based on the Bader charge analysis^51–54^ as follows:

3 $$\Delta \updelta_{{\text{i}}} = \updelta_{{\text{i}}}^{{{\text{ads}}}} - \updelta_{{\text{i}}}^{{{\text{clean}}}}$$

The ${\updelta }_{{\text{i}}}^{{{\text{clean}}}}$ denotes the partial charge of isolated adsorbate i species (i denoted three systems: (1) isolated C atom or C cluster (depending on the size of coke model) and (2) clean catalyst surfaces) and the $\updelta_{{\text{i}}}^{{{\text{ads}}}}$ denotes the partial charge of adsorbed i species after adsorption. For the charge changes analysis, the positive value of the charge transfer is designated as the electron gain, while the negative value indicated electron loss of an atom of interest. The mechanism of C diffusion and the transition state (TS) of each elementary step were determined via the climbing-image nudge-elastic band (CI-NEB)^55^ method, which locates the TS structure. All TS structures were calculated based on each elementary step’s initial and final structures following a standard optimization. Subsequently, all obtained TS structures were confirmed for the saddle point on the potential energy surface by verifying an imaginary vibrational frequency value. The coke movement on the catalyst surface was described via the rate of coke diffusion. The forward rate constant (k_f_) was obtained via the Eyring equation as follows.

4 $${\text{k}}_{{\text{f}}} = \frac{{{\text{k}}_{{\text{B}}} {\text{T}}}}{{\text{h}}}{\text{exp}}\left( {\frac{{ - {\Delta\text{G}}_{{\text{f}}} }}{{{\text{k}}_{{\text{B}}} {\text{T}}}}} \right)$$

The parameter h is the Planck’s constant (4.14 × 10^–15^ eV s^–1^), and k_B_ is the Boltzmann constant (8.617 × 10^–5^ eV K^–1^). The *∆*G_f_ is the activation free energy of a forward reaction, which was calculated from the Gibbs free energy changes from the initial state to its transition state at 1000 K. The Gibbs free energy of each state $\left( {\Delta {\text{G}}_{{\text{i}}} } \right)$ was calculated as follows.

5 $$\Delta {\text{G}}_{{\text{i}}} = {\text{E}}_{{{\text{DFT}}}} + {\text{E}}_{{{\text{ZPE}}}} - {\text{RT}}\ln {\text{Q}}_{{{\text{vib}}}}$$

The term E_DFT_ is the total energy of species i provided from the DFT calculation, R is the gas constant 1.43 × 10^–28^ eV K^–1^ mol^–1^, The Q_vib_ is the total vibrational partition function of the adsorbed system in which the measurement from vibrational analysis and E_ZPE_ is the zero-point energy of the adsorbed system given by the following equation.

6 $$\Delta{\text{E}}_{{{\text{ZPE}}}} = \sum \frac{{{\text{h}}\upnu_{{\text{i}}} }}{2}$$

The term ν_i_ is the vibrational frequency obtained via the vibrational frequency calculation.

#### Coke mobility calculation

The rate of C diffusion at the reaction temperature equal to 1000 K was calculated via the equation below.

7 $${\hat{\text{r}}}_{i} = {\text{k}}_{{\text{f}}} \theta_{{{\text{start}}}} {-}{\text{ k}}_{{\text{r}}} \theta_{{{\text{end}}}}$$

The parameter k_f_ is the forward rate constant of C diffusion on pure surface i, and k_r_ is the reverse rate constant of C diffusion also on the pure surface i. The parameter θ_start_ is the sum of carbon atom surface coverage at the most stable adsorption site of pure surface i, which was designated the starting point of surface diffusion, while the θ_end_ represents the carbon atom surface coverage of the final diffusion site of pure surface i.

For the rate of C diffusion on the system consisting of more than one type of surface, e.g., in the case of NiCo bimetallic system, which comprises three regions of Ni, Co, and NiCo, the weighted rate of C diffusion is used as shown in Eq. (8).

8 $${\overline{\text{r}}}_{{\text{i}}} {\text{ = f}}_{{{\text{Ni}}}} {\hat{\text{r}}}_{{{\text{Ni}}}} {\text{ + f}}_{{{\text{Co}}}} {\hat{\text{r}}}_{{{\text{Co}}}} {\text{ + f}}_{{{\text{NiCo}}}} {\hat{\text{r}}}_{{{\text{NiCo}}}}$$

Regarding Eq. (8), the weighted rate of C diffusion (${\overline{\text{r}}}_{{\text{i}}}$) is constructed from each C diffusion rate on a pure surface that is weighted with the parameter f_i_, the surface fraction. The sum of all surface fractions: f_Ni_, f_Co_, and f_NiCo_ is equal to one, while the rate on the pure surface is calculated from Eq. (7)

## Supplementary Information

Supplementary Information.

## Data Availability

The authors declare that relevant data are within the manuscript.
