# Supplementary material for: Experimental and computational investigation on underlying factors promoting high coke resistance in NiCo bimetallic catalysts during dry reforming of methane
Source: Sci Rep. 2021 Jan 12;11:519. doi: 10.1038/s41598-020-80287-0 (PMC7804276; doi:10.1038/s41598-020-80287-0)
Supplement: Supplementary file 1 — Supplementary Information. [file 41598_2020_80287_MOESM1_ESM.pdf]

Supplementary Document of

Experimental and computational investigation on underlying factors promoting high coke resistance in NiCo bimetallic catalysts during dry reforming of methane

Tinnakorn Saelee[1,2,‡], Mongkol Lerdpongsiripaisarn[1,2,‡], Meena Rittiruam[1,2], Siriwimol Somdee[1,2], Anchittha Liu[2], Supareak Praserthdam[1,2,*], Piyasan Praserthdam[2]

[1]High-Performance Computing Unit (CECC-HCU), Center of Excellence on Catalysis and Catalytic Reaction Engineering (CECC), Chulalongkorn University, Bangkok 10330, Thailand

[2]Center of Excellence on Catalysis and Catalytic Reaction Engineering (CECC), Chulalongkorn University, Bangkok 10330, Thailand

[‡]These authors contributed equally to this work

*Corresponding author's email: supareak.p@chula.ac.th (Dr. Supareak Praserthdam)

List of Tables **Page**

Table S1 possible active sites, coke adsorption energy (E_{ads}), and optimized coke-adsorption height on Ni100, Ni111, Ni211, NiCo100, NiCo111, and NiCo211 surfaces 3

Table S2 Bader charge analysis of C1 coke and coke-adsorbed Ni and NiCo surfaces, the nearest Ni atom (in the case of Ni and NiCo), and the nearest Co atom (in the case of NiCo) on Ni100, Ni111, Ni211, NiCo100, NiCo111, and NiCo211 surfaces 4

Table S3 Bader charge analysis of C2 coke molecule, individual C atoms in C2 coke, and coke-adsorbed Ni and NiCo surfaces, the nearest Ni atom (in the case of Ni and NiCo), and the nearest Co atom (in the case of NiCo) on Ni100, Ni111, Ni211, NiCo100, NiCo111, and NiCo211 surfaces 4

Table S4 Bader charge analysis of C3 coke molecule, individual C atoms in C3 coke, and coke-adsorbed Ni and NiCo surfaces, the nearest Ni atom (in the case of Ni and NiCo), and the nearest Co atom (in the case of NiCo) on Ni100, Ni111, Ni211, NiCo100, NiCo111, and NiCo211 surfaces 4

Table S5 The forward ($E_{a,f}$) and reverse ($E_{a,r}$) activation energy of the C atom diffusion and its imaginary frequency of Ni111, Ni211, Co001, NiCo111 and, NiCo211 surfaces 5

Table S6 forward (k_f) and reverse (k_r) rate constants of C atom diffusion for the preferable pathway on Ni111, Co001, and NiCo111 surfaces 5

Table of Figures **Page**

Figure S 1 The TGA (left) and derivative weight loss (right) profiles of coke on pure Ni, pure Co and NiCo catalysts 6
Figure S 2 (a) Surface slab structure of Co001 and possible active sites of the Co001 surface namely (b) HCP and (c) 3-Fold sites 6
Figure S 3 TCP coke diffusion of the 1st stage at reaction coordinate 0 7
Figure S 4 TCP coke diffusion of the 1st stage at reaction coordinate 1 7
Figure S 5 TCP coke diffusion of the 1st stage at reaction coordinate 2 8
Figure S 6 TCP coke diffusion of the 1st stage at reaction coordinate 3 8
Figure S 7 TCP coke diffusion of the 1st stage at reaction coordinate 4 9
Figure S 8 TCP coke diffusion of the 1st stage at reaction coordinate 5 9
Figure S 9 TCP coke diffusion of the 1st stage at reaction coordinate 6 10
Figure S 10 TCP coke diffusion of the 1st stage at reaction coordinate 7 10
Figure S 11 TCP coke diffusion of the 1st stage at reaction coordinate 8 11
Figure S 12 TCP coke diffusion of the 1st stage at reaction coordinate 9 11
Figure S 14 TCP coke diffusion of the 1st stage at reaction coordinate 10 12
Figure S 15 TCP coke diffusion of the 2nd stage at reaction coordinate 10 13
Figure S 16 TCP coke diffusion of the 2nd stage at reaction coordinate 11 13
Figure S 17 TCP coke diffusion of the 2nd stage at reaction coordinate 12 14
Figure S 18 TCP coke diffusion of the 2nd stage at reaction coordinate 13 14
Figure S 19 TCP coke diffusion of the 2nd stage at reaction coordinate 14 15
Figure S 20 TCP coke diffusion of the 2nd stage at reaction coordinate 15 15
Figure S 21 TCP coke diffusion of the 2nd stage at reaction coordinate 16 16
Figure S 22 TCP coke diffusion of the 2nd stage at reaction coordinate 17 16
Figure S 23 TCP coke diffusion of the 2nd stage at reaction coordinate 18 17
Figure S 24 TCP coke diffusion of the 2nd stage at reaction coordinate 19 17
Figure S 25 TCP coke diffusion of the 2nd stage at reaction coordinate 20 18
Figure S 26 TCP coke diffusion of the 2nd stage at reaction coordinate 21 18
Figure S 27 TCP coke diffusion of the 2nd stage at reaction coordinate 22 19
Figure S 28 TCP coke diffusion of the 2nd stage at reaction coordinate 23 19
Figure S 29 TCP coke diffusion of the 2nd stage at reaction coordinate 24 20
Figure S 30 TCP coke diffusion of the 2nd stage at reaction coordinate 25 20
Figure S 31 TCP coke diffusion of the 3rd stage at reaction coordinate 25 21
Figure S 32 TCP coke diffusion of the 3rd stage at reaction coordinate 26 21
Figure S 33 TCP coke diffusion of the 3rd stage at reaction coordinate 27 22
Figure S 34 TCP coke diffusion of the 3rd stage at reaction coordinate 28 22
Figure S 35 TCP coke diffusion of the 3rd stage at reaction coordinate 29 23
Figure S 36 TCP coke diffusion of the 3rd stage at reaction coordinate 30 23

Table S1 possible active sites, coke adsorption energy (E_{ads}), and optimized coke-adsorption height on Ni100, Ni111, Ni211, NiCo100, NiCo111, and NiCo211 surfaces

Catalyst surface	Coke	Adsorption site	E_{ads} (eV)	Adsorption height (Å)
**Ni100**	C1	4-Fold	-9.60*	0.26
	C2	4-Fold	-8.20*	1.08
		Bridge and 4-Fold	-7.92	0.86
	C3	4-Fold and Bridge	-6.93	0.78
		4-Fold (cyclic C3)	-5.17	0.25
		4-Fold	-7.26*	0.81
**Ni111**	C1	HCP	-8.25*	0.96
		FCC	-8.14	0.98
	C2	HCP and FCC	-7.80*	1.33
	C3	HCP and FCC	-5.53	1.30
		FCC and FCC	-5.98*	1.19
		HCP and HCP	-5.92	1.19
**Ni211**	C1	3-Fold	-8.13	0.98
		4-Fold	-9.22*	0.25
	C2	4-Fold and 3-Fold	-8.84*	1.11
	C3	Bridge to Bridge	-6.61	1.41
		4-Fold to 4-Fold	-7.60*	1.01
**NiCo100**	C1	4-Fold	-9.49*	0.31
**NiCo100**	C2	4-Fold	-7.97*	1.16
		Bridge and 4-Fold	-7.92	0.91
	C3	4-Fold (two sites)	-7.39*	0.81
		4-Fold (cyclic C3)	-5.31	0.36
		4-Fold	-5.79	1.36
**NiCo111**	C1	HCP-NiCoCo	-8.26*	0.94
		FCC (NiCoCo)	-8.10	0.95
		HCP-NiNiCo	-8.16	0.96
		FCC-NiNiCo	-8.04	0.99
	C2	FCC-NiCoCo and HCP-NiCoCo	-7.87*	1.26
		HCP-NiCoCo and FCC-NiNiCo	-7.81	1.36
	C3	FCC-NiCoCo and FCC-NiNiCo	-5.88*	1.17
		FCC-NiCoCo and FCC-NiCoCo	-5.58	1.33
		FCC-NiNiCo and FCC-NiNiCo	-5.71	1.20
**NiCo211**	C1	3-Fold-NiCoCo	-8.15	0.98
		3-Fold-NiNiCo	-8.22	0.92
		4-Fold-NiNiNiNi	-8.96*	0.26
		Bridge-CoCo	-7.99	1.20
	C2	4-Fold-NiNiNiNi and 3-Fold NiNiCo	-8.77*	1.15
	C3	4-Fold- NiNiNiNi and Bridge-CoCo	-7.41*	1.12
		Bridge-CoCo and 3-Fold NiNiCo	-6.34	1.25

*The most stable adsorption site

Table S2 Bader charge analysis of C1 coke and coke-adsorbed Ni and NiCo surfaces, the nearest Ni atom (in the case of Ni and NiCo), and the nearest Co atom (in the case of NiCo) on Ni100, Ni111,

Surface	E_{ads}	Bader charge of total C1	Bader charge of Nearest-Ni atoms	Bader charge of Nearest-Co atoms	Bader charge of Nearest-NiCo cluster
Ni100	-9.60	-0.77	+2.13	-	+2.13
Ni111	-8.25	-0.11	+0.34	-	+0.34
Ni211	-9.22	-0.16	+0.93	-	+0.93
NiCo100	-9.49	+0.05	-0.19	+0.52	+0.35
NiCo111	-8.26	+0.37	+0.08	-0.34	-0.26
NiCo211	-8.96	+0.16	+1.19	-0.45	0.74

Ni211, NiCo100, NiCo111, and NiCo211 surfaces

Table S3 Bader charge analysis of C2 coke molecule, individual C atoms in C2 coke, and coke-adsorbed Ni and NiCo surfaces, the nearest Ni atom (in the case of Ni and NiCo), and the nearest Co atom (in the case of NiCo) on Ni100, Ni111, Ni211, NiCo100, NiCo111, and NiCo211 surfaces

Surface	E_{ads}	Bader charge of C2			Bader charge of Nearest-Ni atoms	Bader charge of Nearest-Co atoms	Bader charge of Nearest-NiCo cluster
		C2_1	C2_1	Total C2			
Ni100	-8.20	+0.42	-0.24	+0.19	-0.75	-	-0.75
Ni111	-7.80	+0.19	+0.49	+0.69	-0.76	-	-0.76
Ni211	-8.84	+0.48	+0.36	+0.85	-0.49	-	-0.49
NiCo100	-8.25	+0.85	-0.11	+0.73	-0.39	-0.14	-0.53
NiCo111	-7.87	+0.21	+0.20	+0.41	-0.10	-0.33	-0.43
NiCo211	-8.77	+0.72	+0.29	+1.01	-0.09	-0.29	-0.38

Table S4 Bader charge analysis of C3 coke molecule, individual C atoms in C3 coke, and coke-adsorbed Ni and NiCo surfaces, the nearest Ni atom (in the case of Ni and NiCo), and the nearest Co atom (in the case of NiCo) on Ni100, Ni111, Ni211, NiCo100, NiCo111, and NiCo211 surfaces

Surface	E_{ads}	Bader charge of C3				Bader charge of Nearest-Ni atoms	Bader charge of Nearest-Co atoms	Bader charge of Nearest-NiCo atoms
		C3_1	C3_2	C3_3	Total C3			
Ni100	-7.26	+0.20	-0.06	+0.57	+0.71	-0.40	-	-0.40
Ni111	-5.98	-0.06	+0.38	+0.14	+0.46	-0.43	-	-0.43
Ni211	-7.60	-0.38	+0.71	+0.42	+0.75	-0.33	-	-0.33
NiCo100	-7.39	+0.39	+0.06	+0.74	+1.19	-0.41	-0.83	-1.24
NiCo111	-5.88	-0.34	+0.56	+0.34	+0.57	-0.35	-0.17	-0.52
NiCo211	-7.41	-0.48	+0.42	+0.90	+0.84	-0.07	-1.06	-0.09

Table S5 The forward ($E_{a,f}$) and reverse ($E_{a,r}$) activation energy of the C atom diffusion and its imaginary frequency of Ni111, Ni211, Co001, NiCo111 and, NiCo211 surfaces

Catalyst Surface	Elementary step	$E_{a,f}$ (eV)	$E_{a,r}$ (eV)	Imaginary freq. (cm^{-1})
**Ni111**	C_{HCP} → C_{FCC}	0.50	0.39	-304.51
**Ni211**	$C_{4\text{-}Fold}$ → $C_{3\text{-}Fold}$	1.42	0.34	-285.64
**Co001**	C_{HCP} → $C_{3\text{-}Fold}$	0.42	0.19	-198.40
**NiCo111**	C_{HCPNi1} → C_{FCCNi1}	0.34	0.19	-270.42
	C_{HCPNi2} → C_{FCCNi1}	0.43	0.36	-258.72
	C_{HCPNi1} → C_{FCCNi2}	0.51	0.30	-278.92
	C_{HCPNi2} → C_{FCCNi2}	0.69	0.57	-315.64
	C_{FCCNi1} → C_{FCCNi2}	0.36	0.30	-281.36
	C_{HCPNi1} → C_{HCPNi2}	0.52	0.43	-253.93
**NiCo211**	$C_{4\text{-}Fold}$ → $C_{3\text{-}FoldNi1}$	1.10	0.30	-155.15
	$C_{4\text{-}Fold}$ → $C_{3\text{-}FoldNi2}$	1.01	0.26	-199.41
	$C_{3\text{-}FoldNi2}$ → $C_{3\text{-}FoldNi1}$	0.34	0.27	-195.15

Table S6 forward (k_f) and reverse (k_r) rate constants of C atom diffusion for the preferable pathway on Ni111, Co001, and NiCo111 surfaces

Catalyst surface	Elementary step	k_f	k_r
Ni111	C_{HCP} → C_{FCC}	$3.71x10^{10}$	$5.49\ x10^{10}$
Co001	C_{HCP} → $C_{3\text{-}Fold}$	$4.88\ x10^{10}$	$4.00\ x10^{10}$
NiCo111	$C_{4\text{-}Fold}$ → $C_{3\text{-}FoldNi2}$	$5.76\ x10^{11}$	$1.17\ x10^{12}$

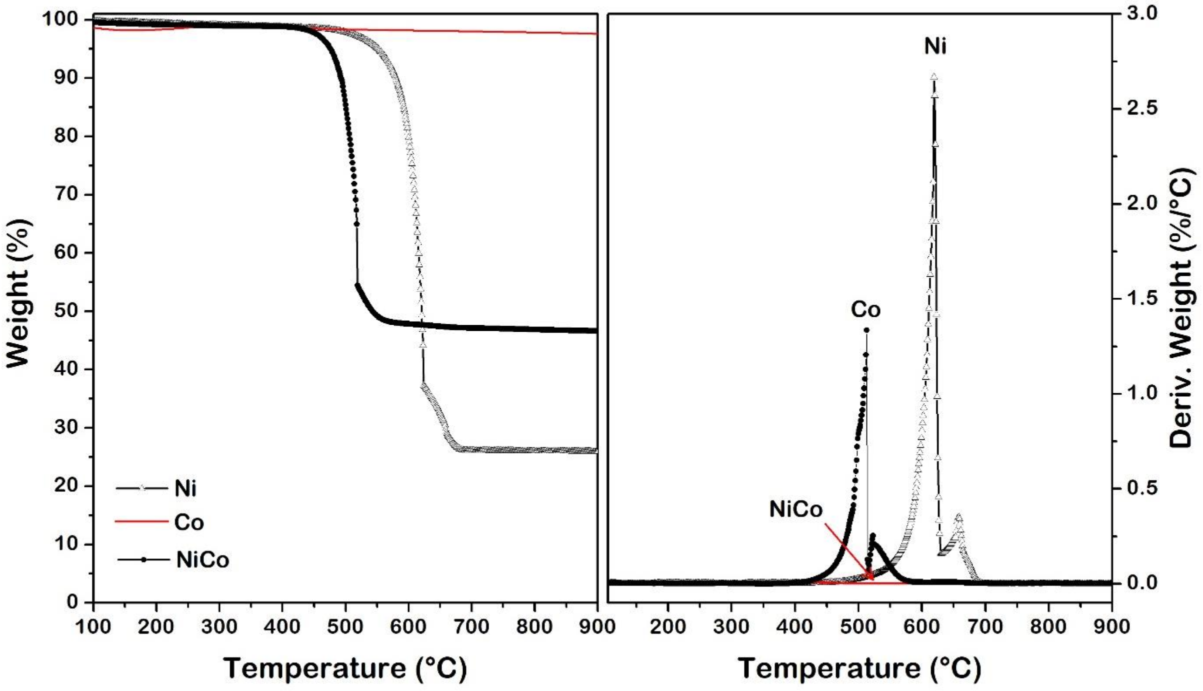

Figure S 1 The TGA (left) and derivative weight loss (right) profiles of coke on pure Ni, pure Co and NiCo catalysts

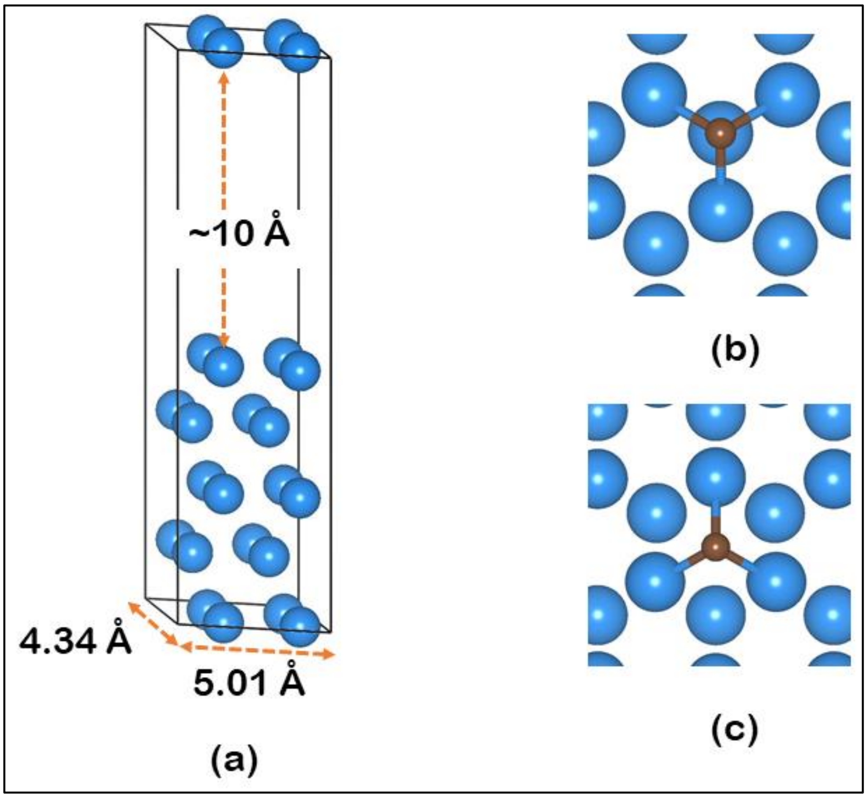

Figure S 2 (a) Surface slab structure of Co001 and possible active sites of the Co001 surface namely (b) HCP and (c) 3-Fold sites

1st Stage Carbon Diffusion

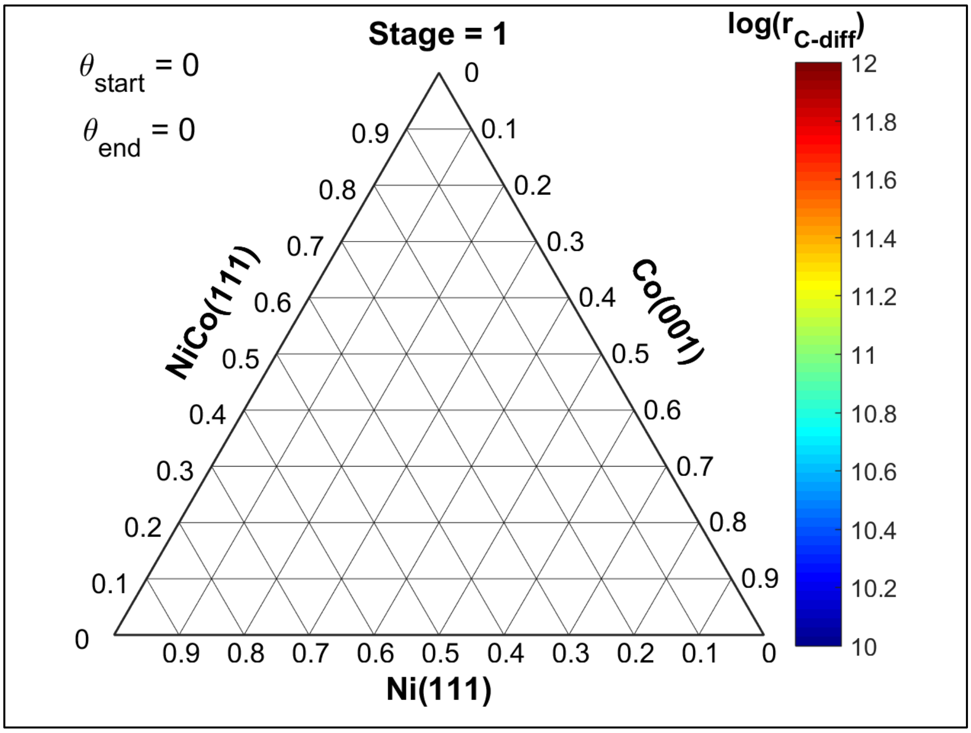

Figure S 3 TCP coke diffusion of the 1st stage at reaction coordinate 0

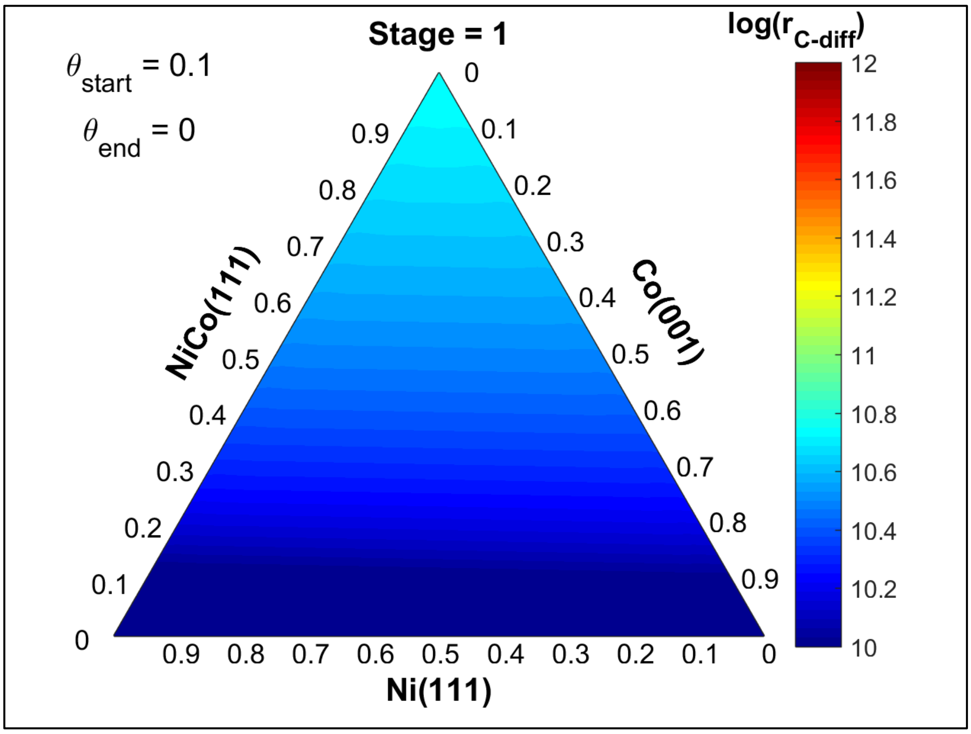

Figure S 4 TCP coke diffusion of the 1st stage at reaction coordinate 1

1st Stage Carbon Diffusion (continued)

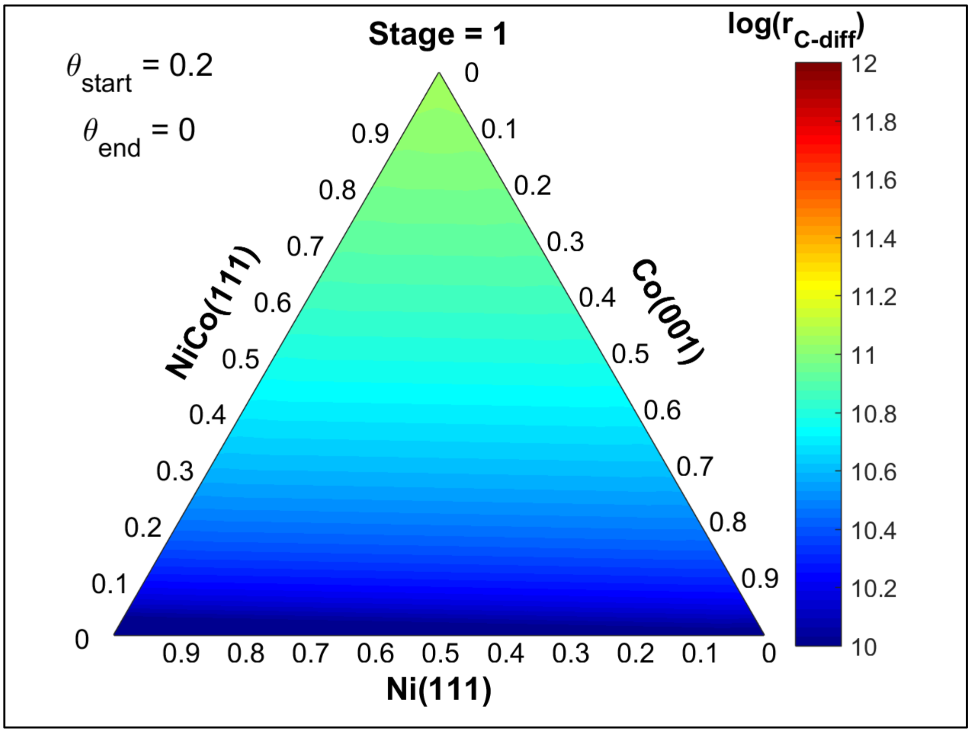

Figure S 5 TCP coke diffusion of the 1st stage at reaction coordinate 2

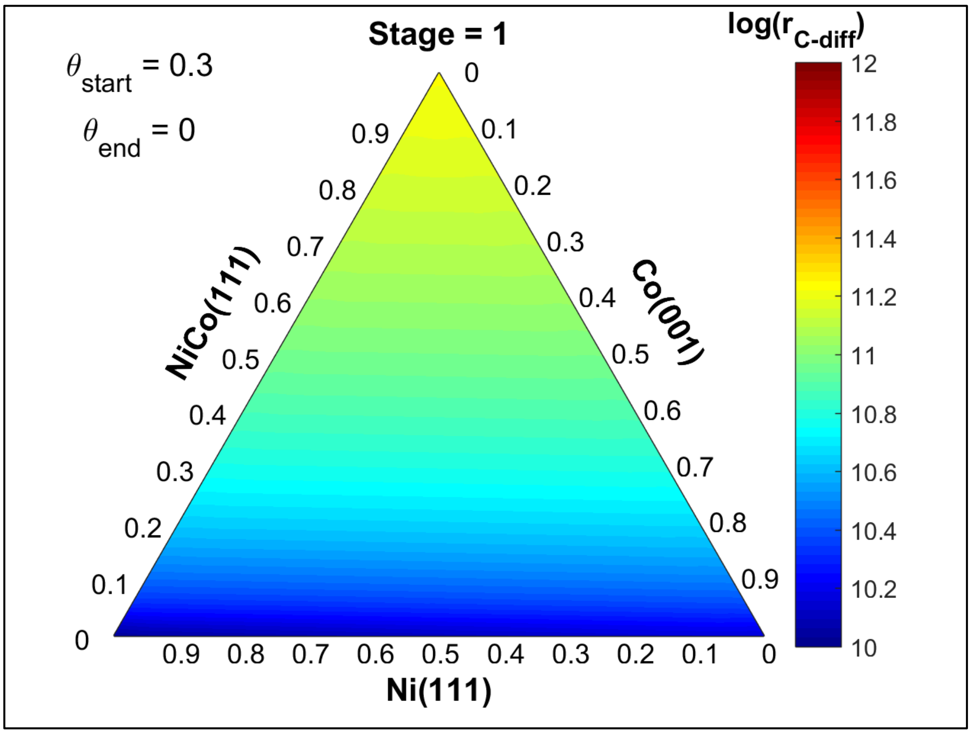

Figure S 6 TCP coke diffusion of the 1st stage at reaction coordinate 3

<u>**1st Stage Carbon Diffusion (continued)**</u>

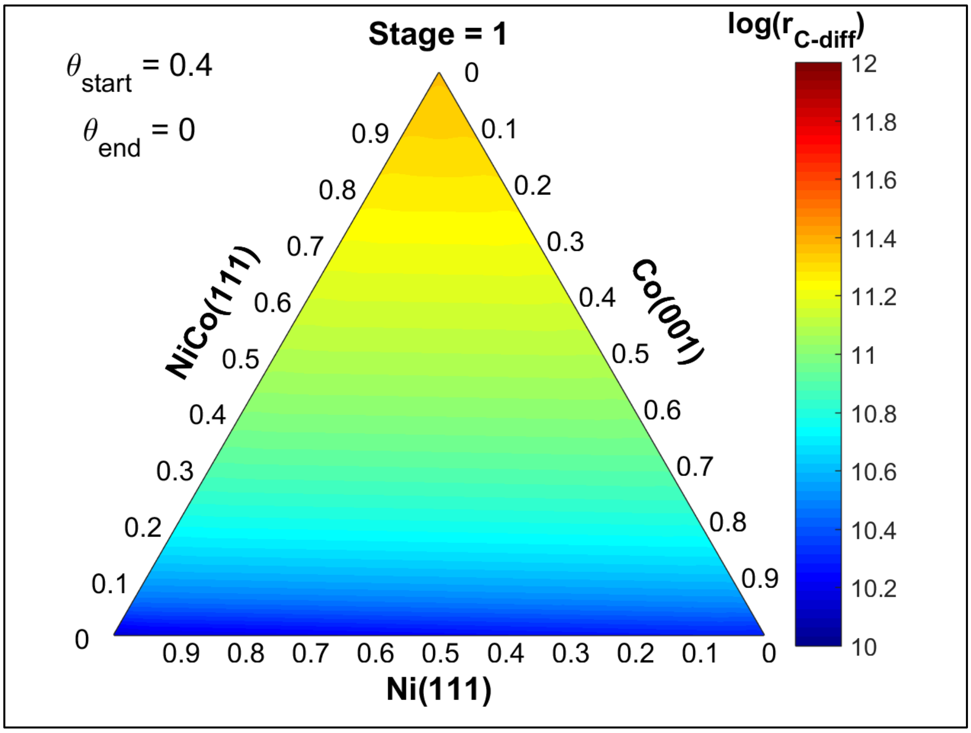

Figure S 7 TCP coke diffusion of the 1st stage at reaction coordinate 4

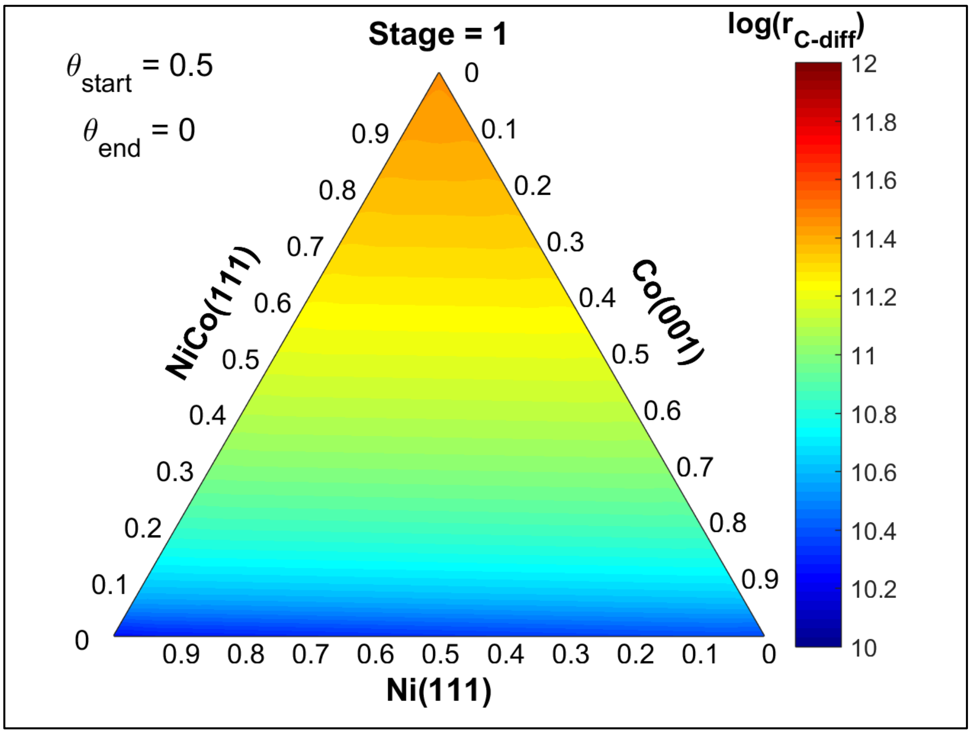

Figure S 8 TCP coke diffusion of the 1st stage at reaction coordinate 5

1st Stage Carbon Diffusion (continued)

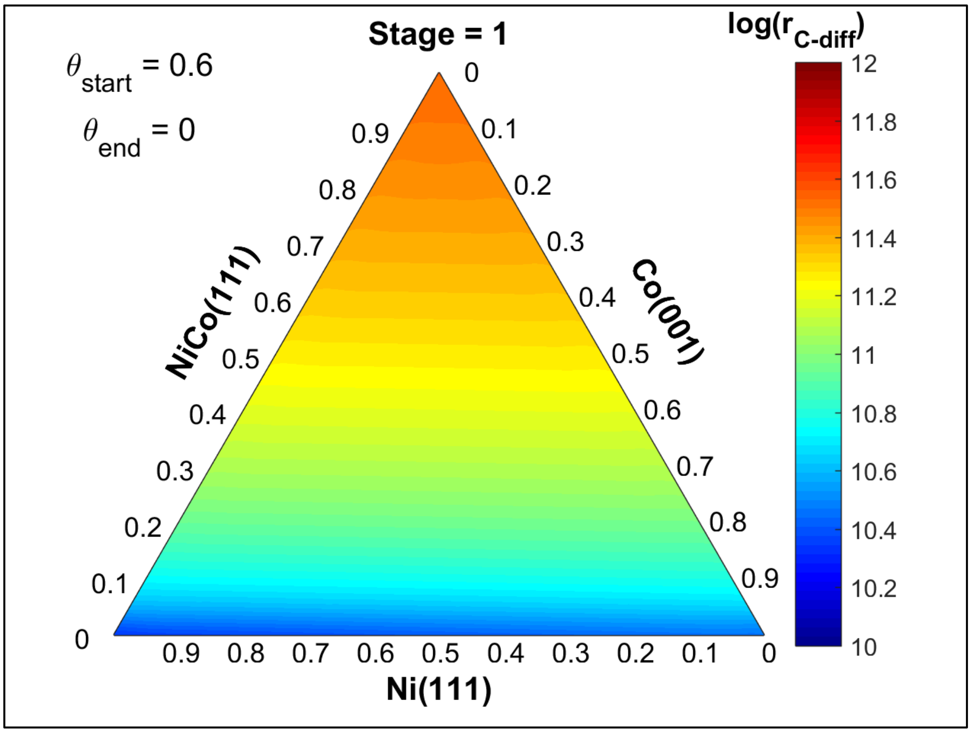

Figure S 9 TCP coke diffusion of the 1st stage at reaction coordinate 6

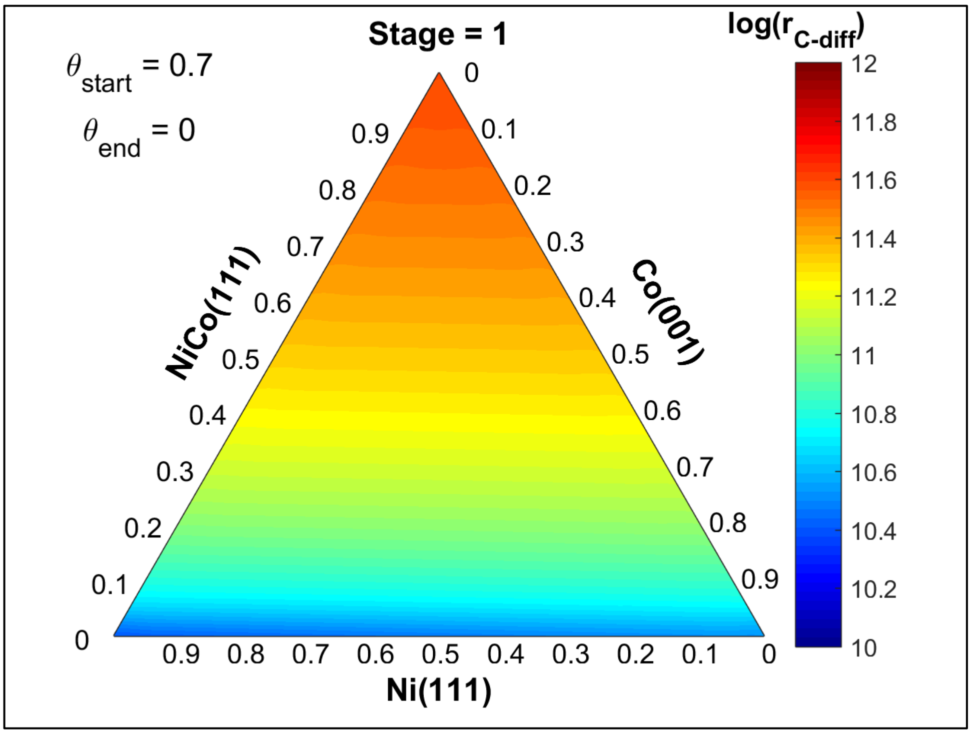

Figure S 10 TCP coke diffusion of the 1st stage at reaction coordinate 7

1st Stage Carbon Diffusion (continued)

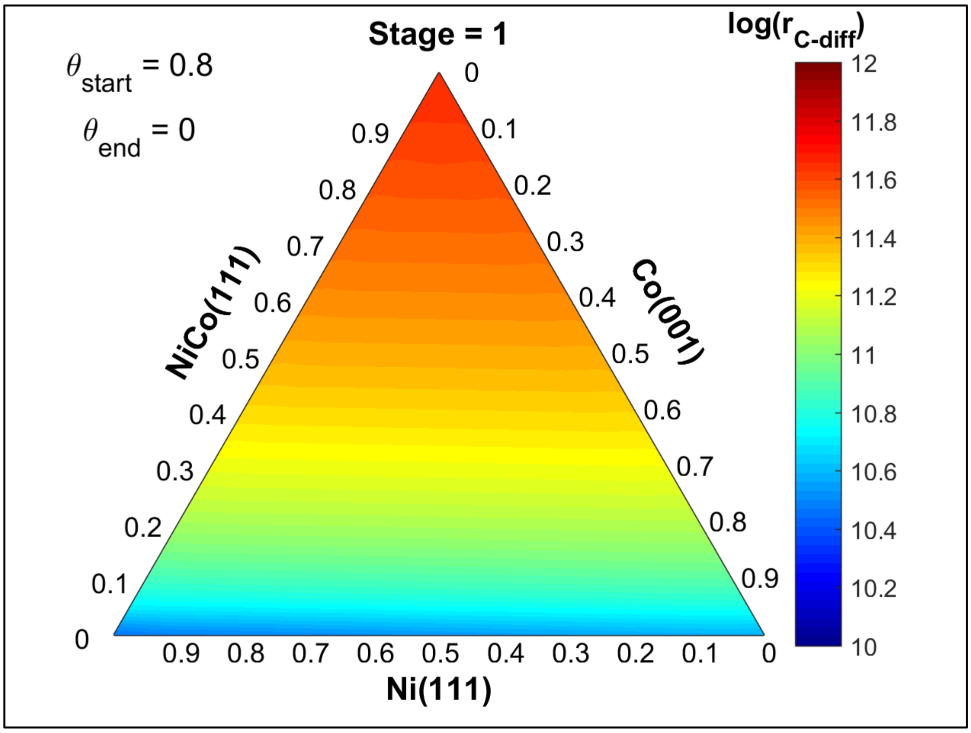

Figure S 11 TCP coke diffusion of the 1st stage at reaction coordinate 8

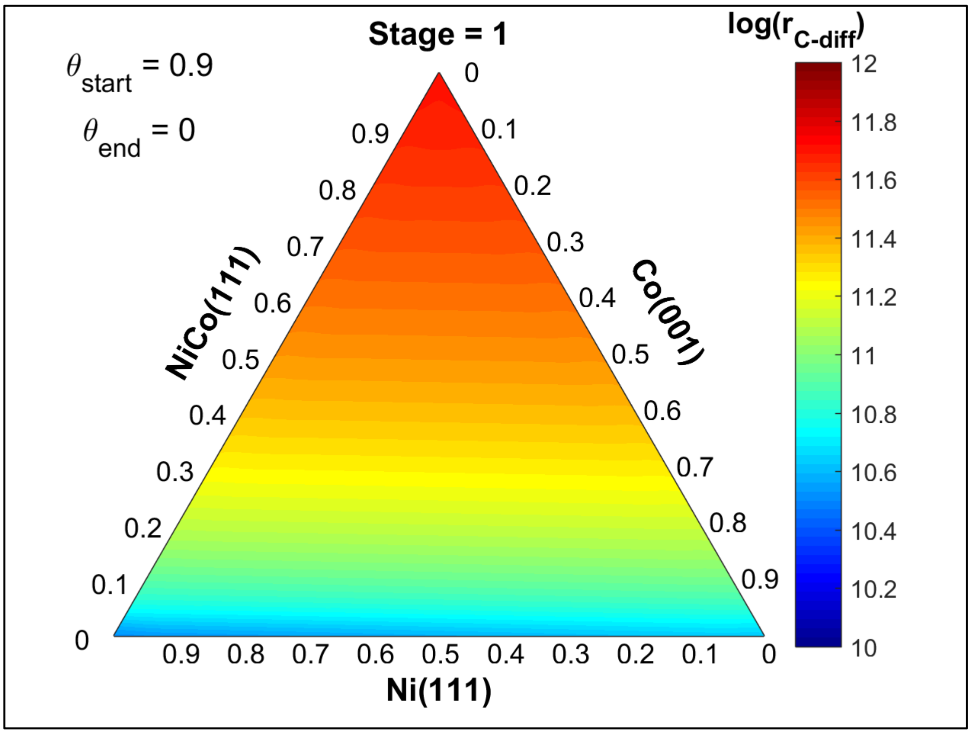

Figure S 12 TCP coke diffusion of the 1st stage at reaction coordinate 9

1st Stage Carbon Diffusion (continued)

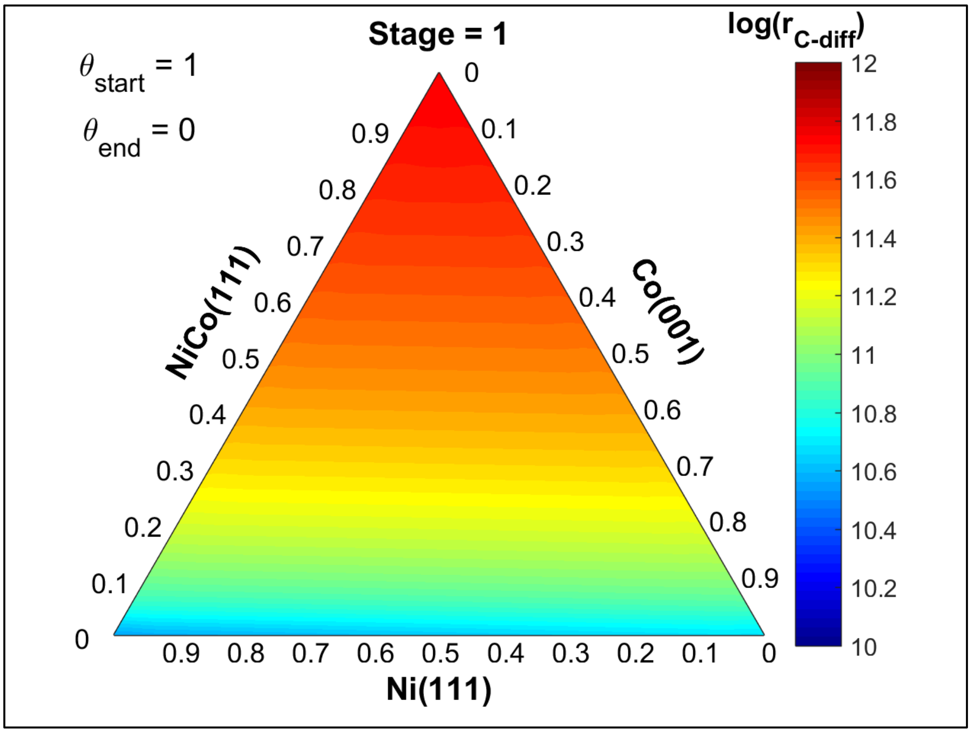

Figure S 13 TCP coke diffusion of the 1st stage at reaction coordinate 10

2nd Stage Carbon Diffusion

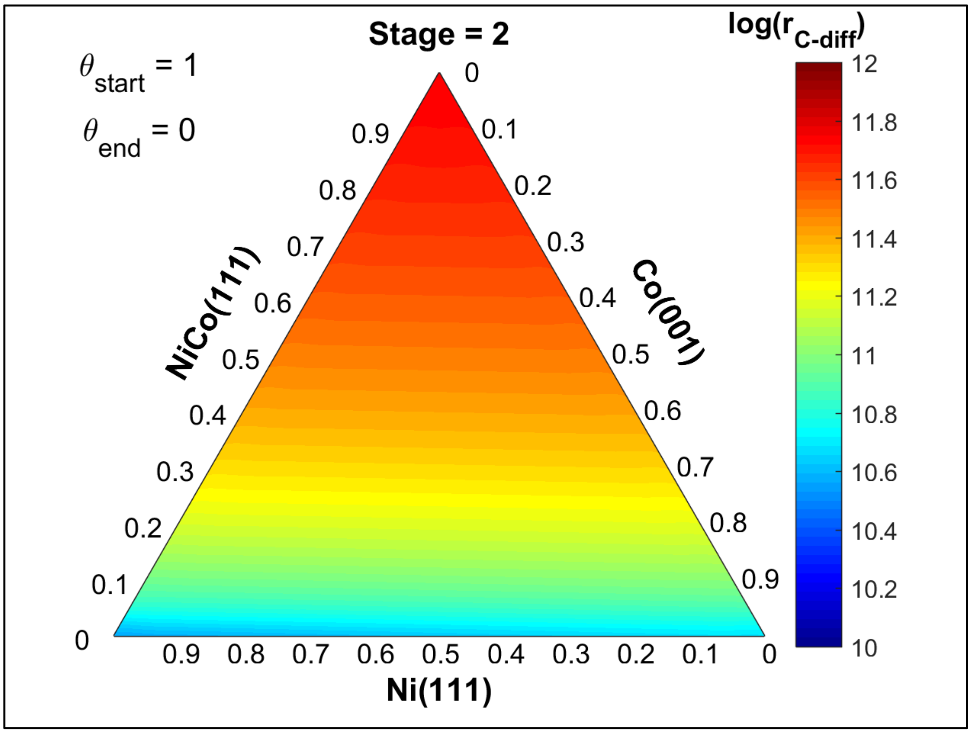

Figure S 14 TCP coke diffusion of the 2nd stage at reaction coordinate 10

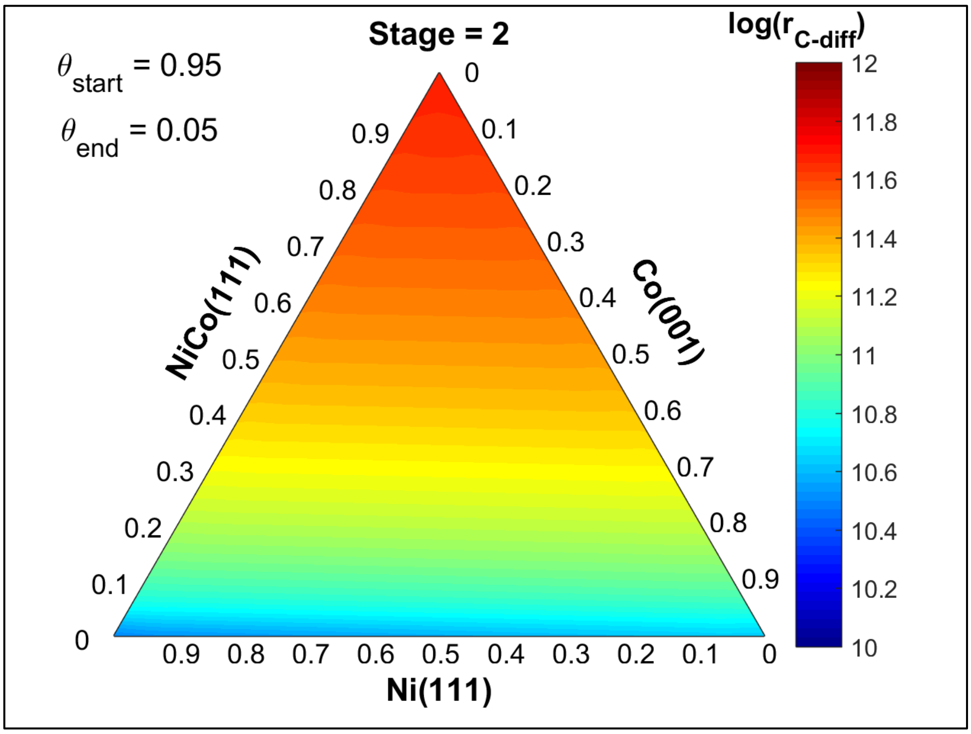

Figure S 15 TCP coke diffusion of the 2nd stage at reaction coordinate 11

2nd Stage Carbon Diffusion (continued)

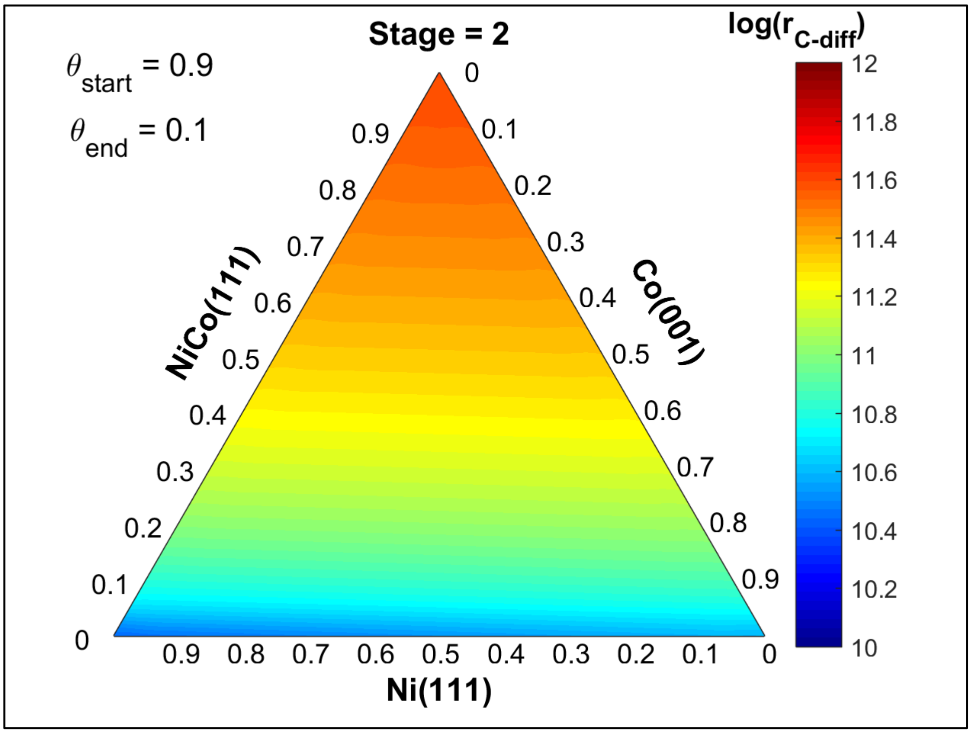

Figure S 16 TCP coke diffusion of the 2nd stage at reaction coordinate 12

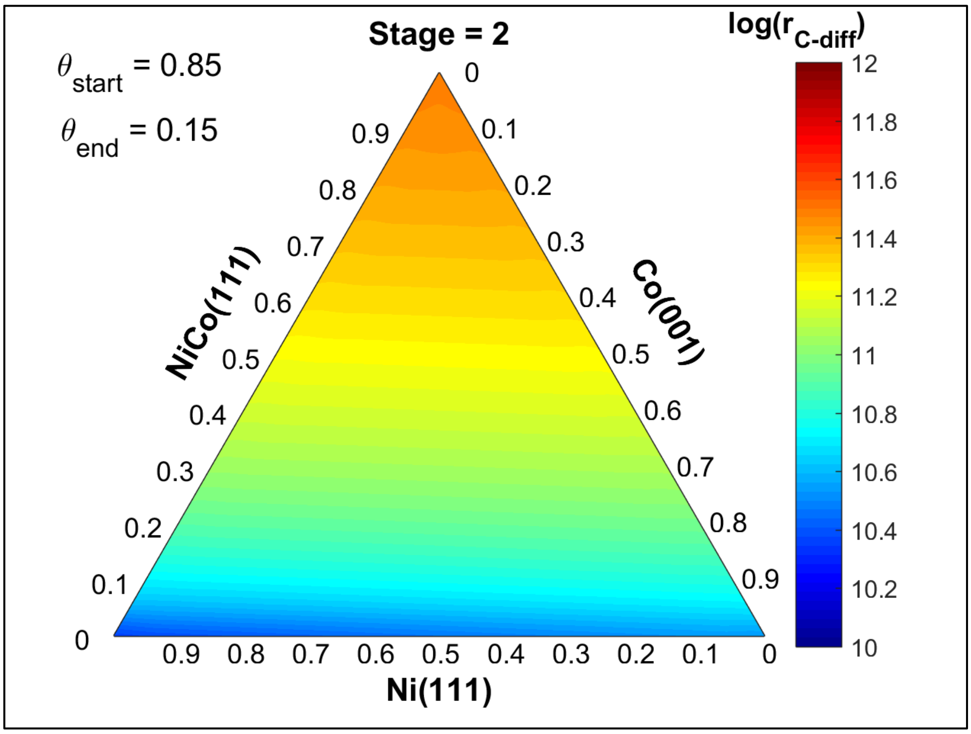

Figure S 17 TCP coke diffusion of the 2nd stage at reaction coordinate 13

2nd Stage Carbon Diffusion (continued)

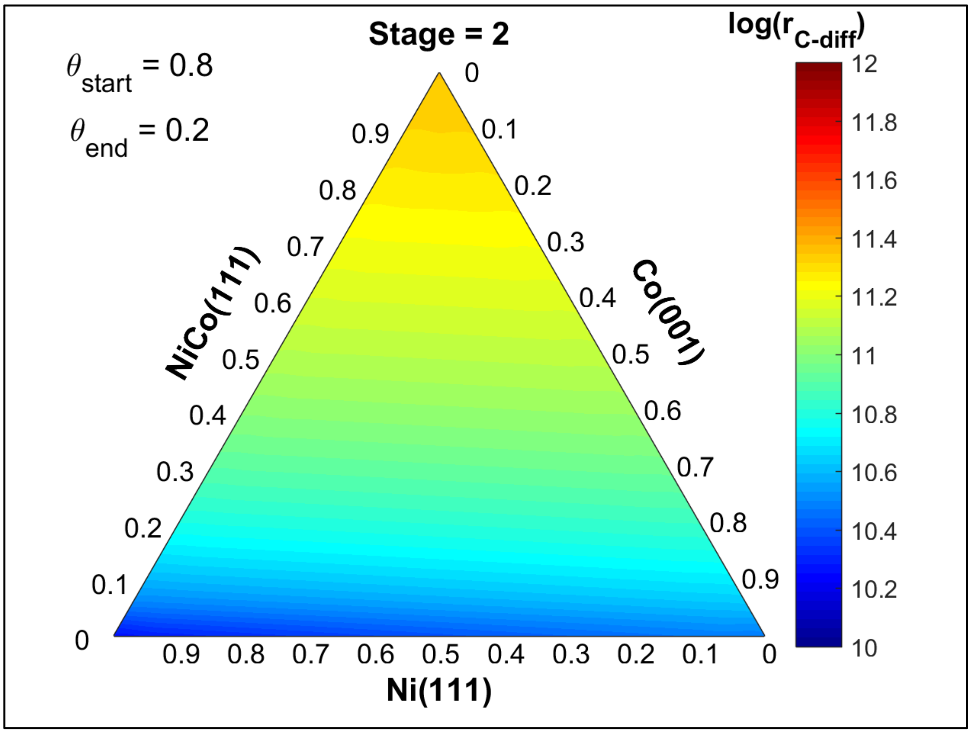

Figure S 18 TCP coke diffusion of the 2nd stage at reaction coordinate 14

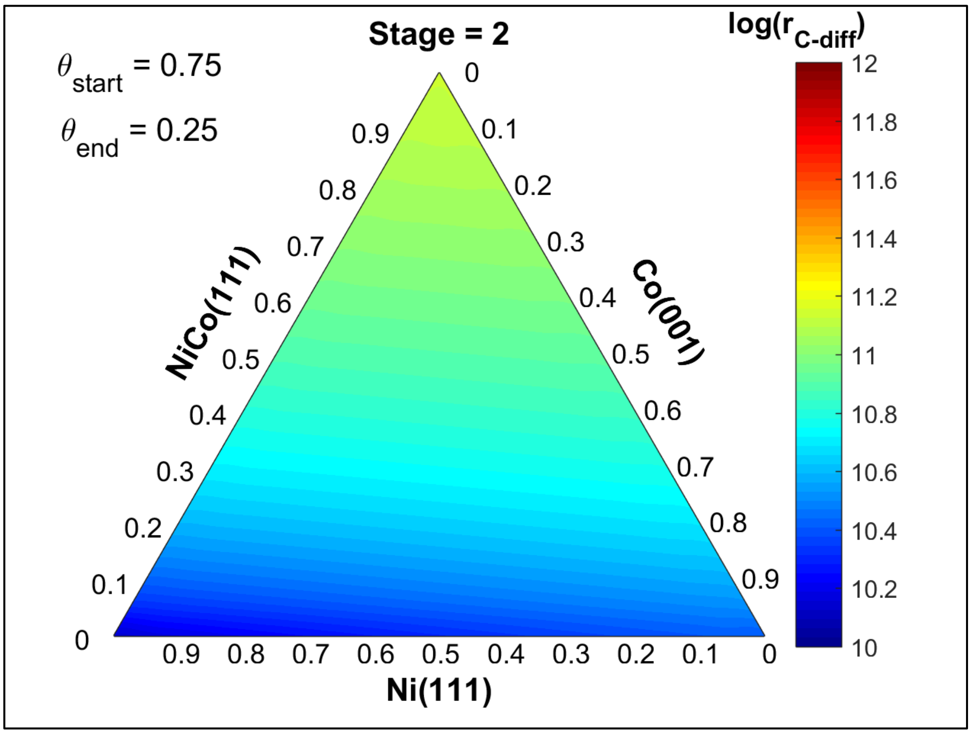

Figure S 19 TCP coke diffusion of the 2nd stage at reaction coordinate 15

2nd Stage Carbon Diffusion (continued)

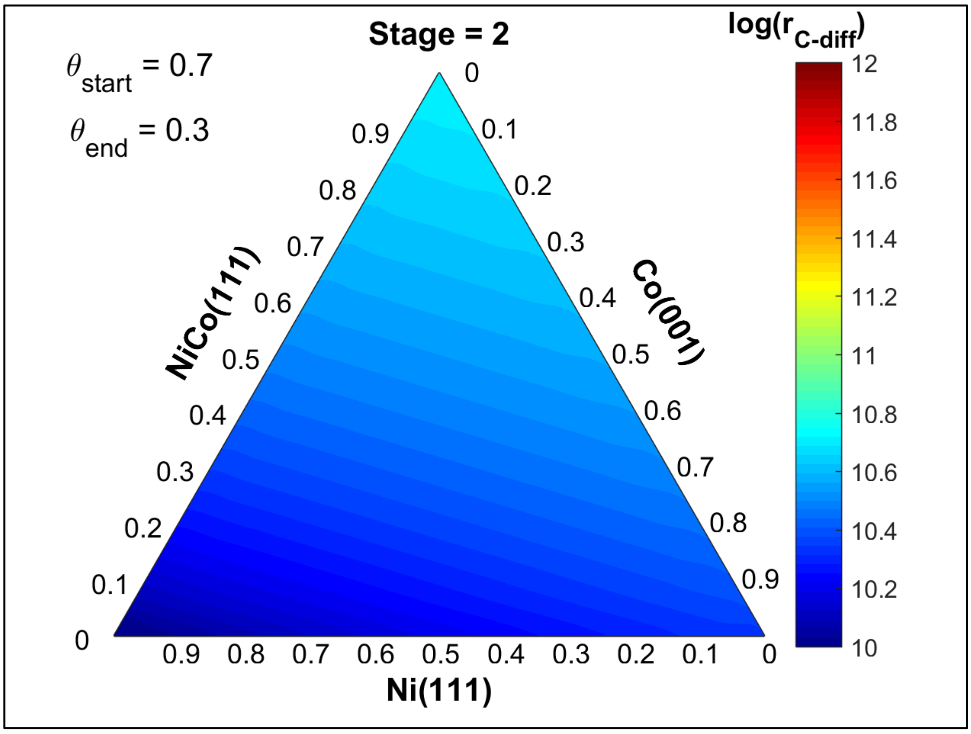

Figure S 20 TCP coke diffusion of the 2nd stage at reaction coordinate 16

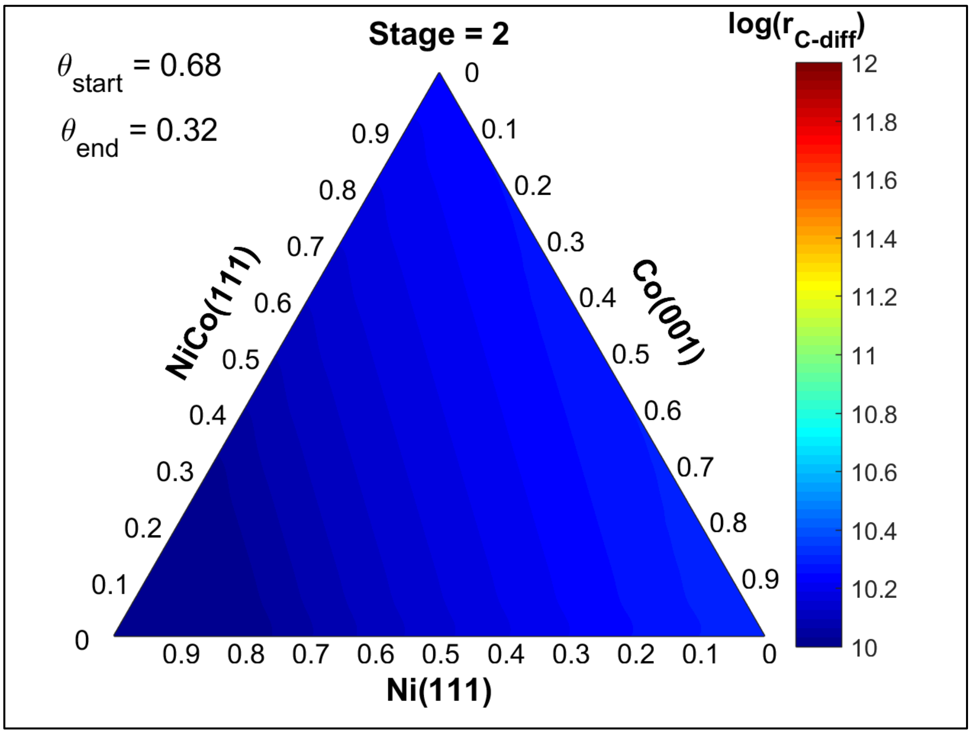

Figure S 21 TCP coke diffusion of the 2nd stage at reaction coordinate 17

2nd Stage Carbon Diffusion (continued)

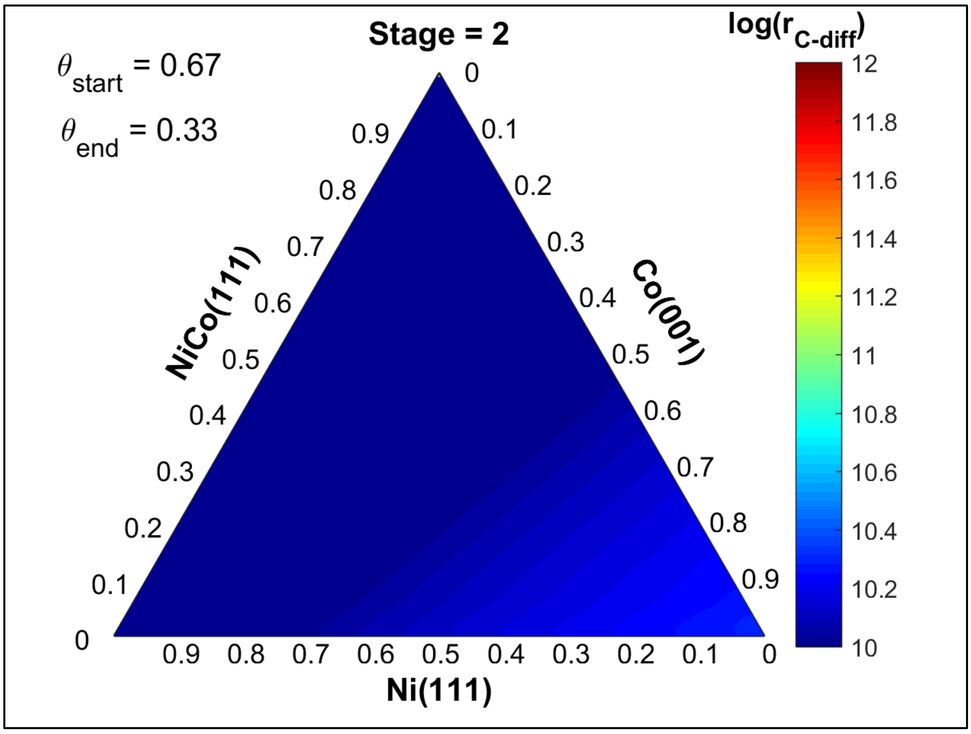

Figure S 22 TCP coke diffusion of the 2nd stage at reaction coordinate 18

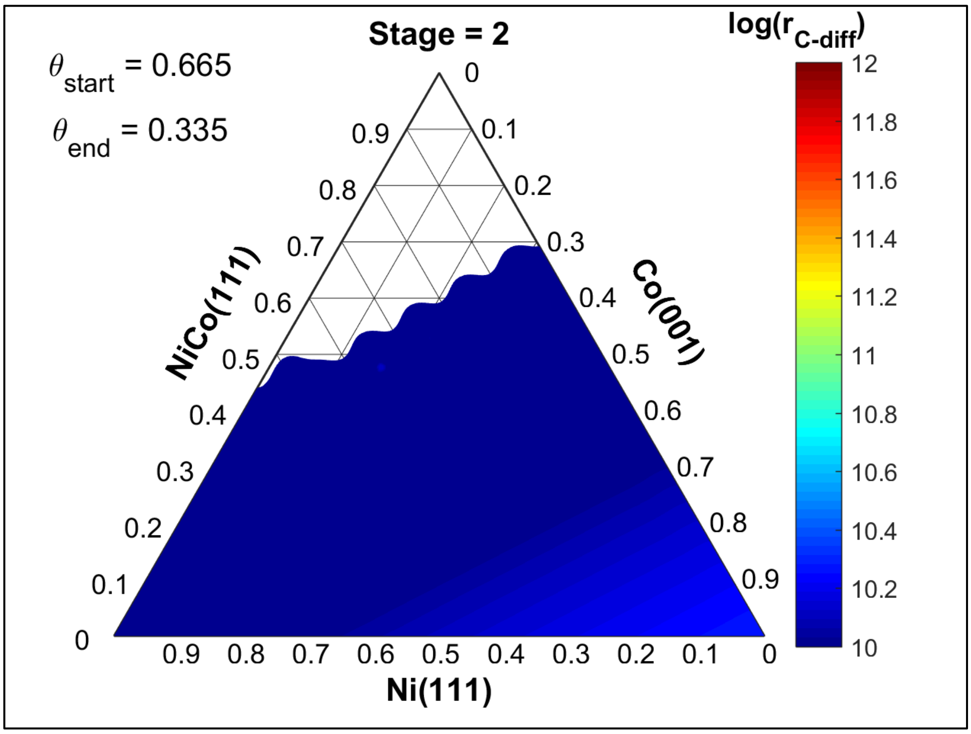

Figure S 23 TCP coke diffusion of the 2nd stage at reaction coordinate 19

2nd Stage Carbon Diffusion (continued)

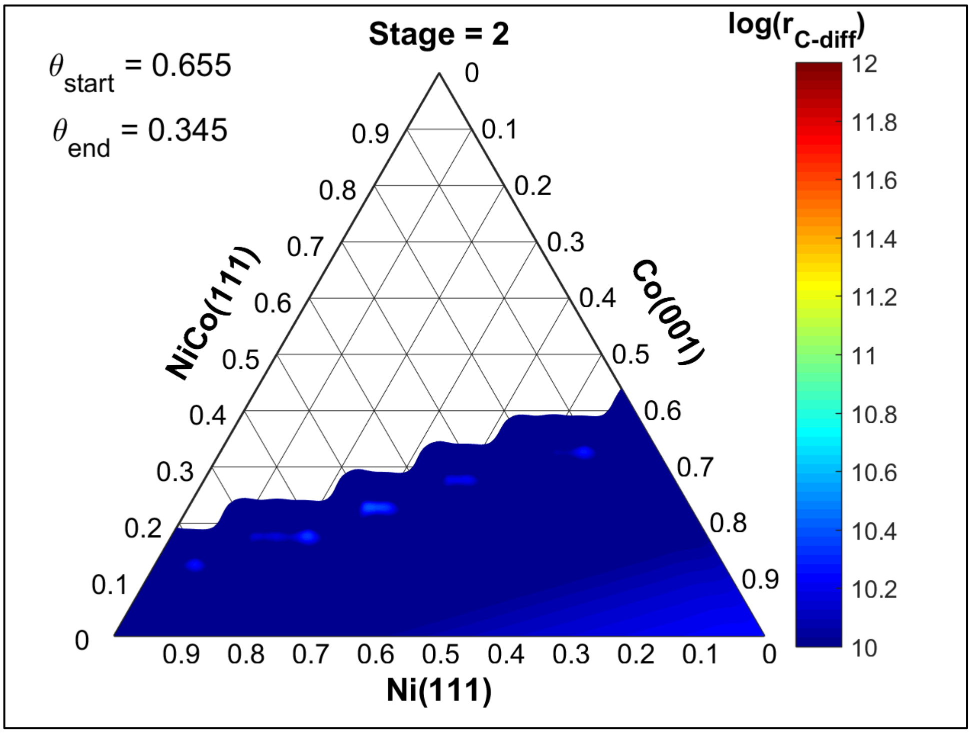

Figure S 24 TCP coke diffusion of the 2nd stage at reaction coordinate 20

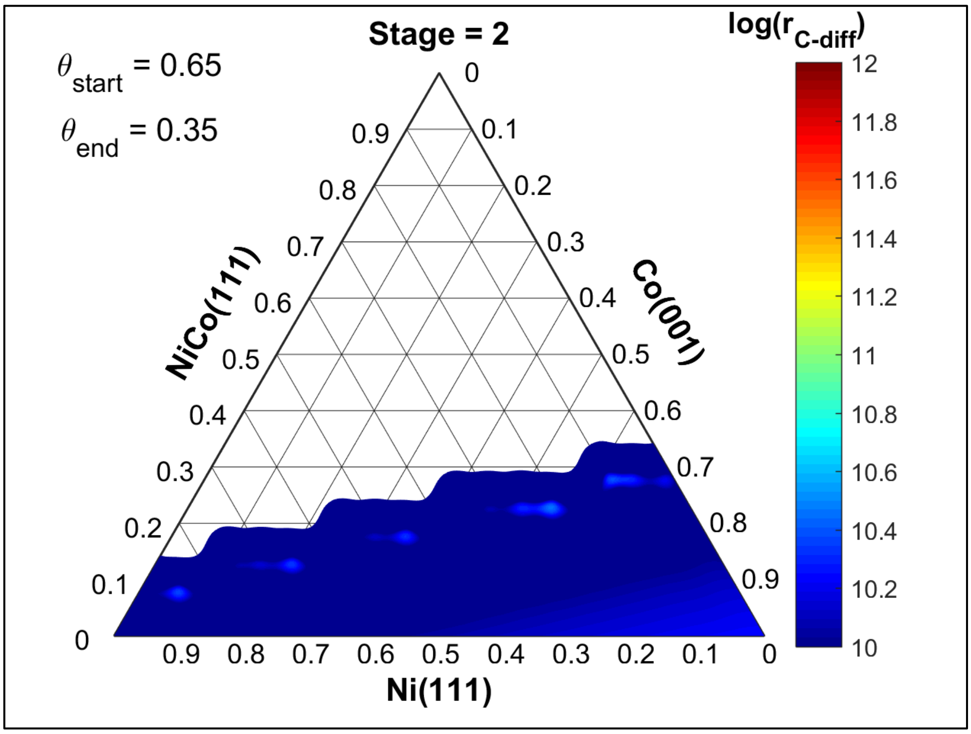

Figure S 25 TCP coke diffusion of the 2nd stage at reaction coordinate 21

2nd Stage Carbon Diffusion (continued)

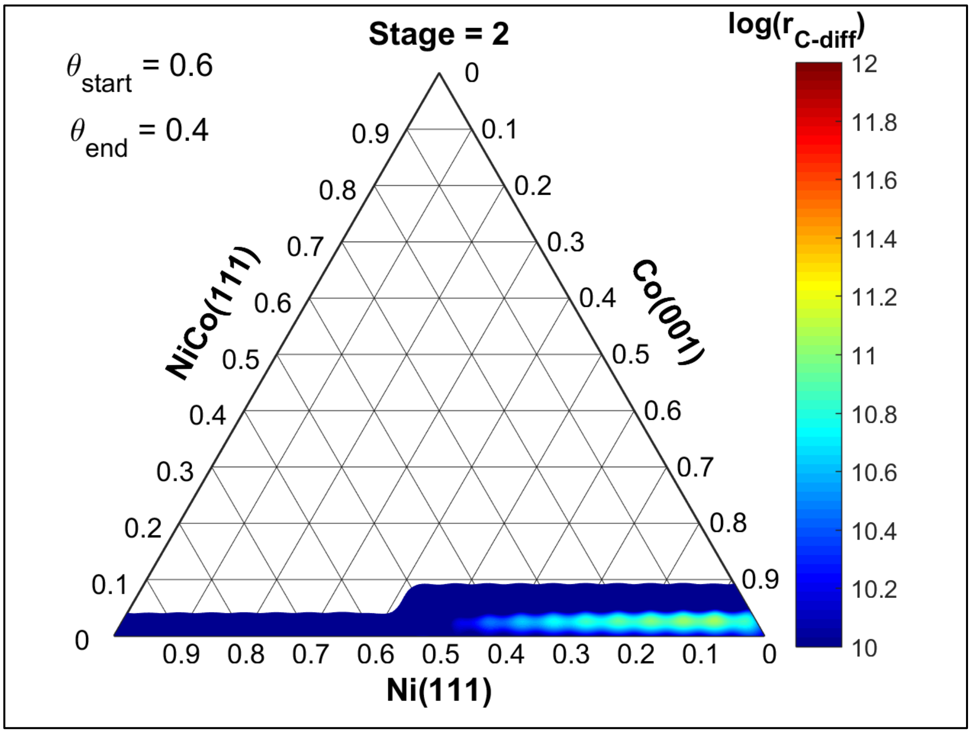

Figure S 26 TCP coke diffusion of the 2nd stage at reaction coordinate 22

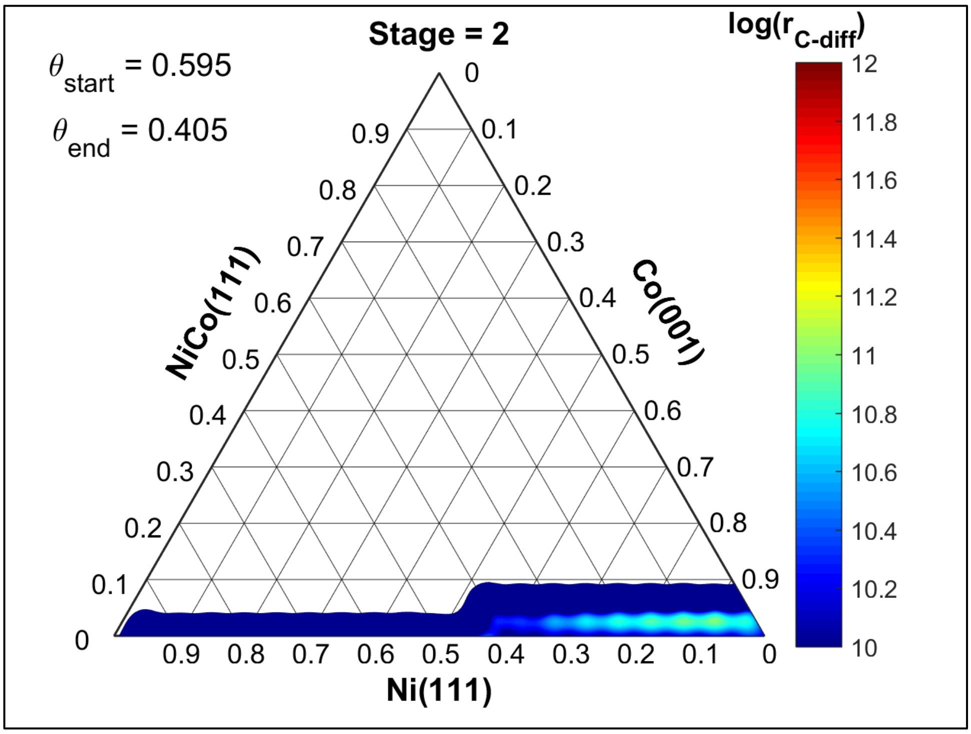

Figure S 27 TCP coke diffusion of the 2nd stage at reaction coordinate 23

2nd Stage Carbon Diffusion (continued)

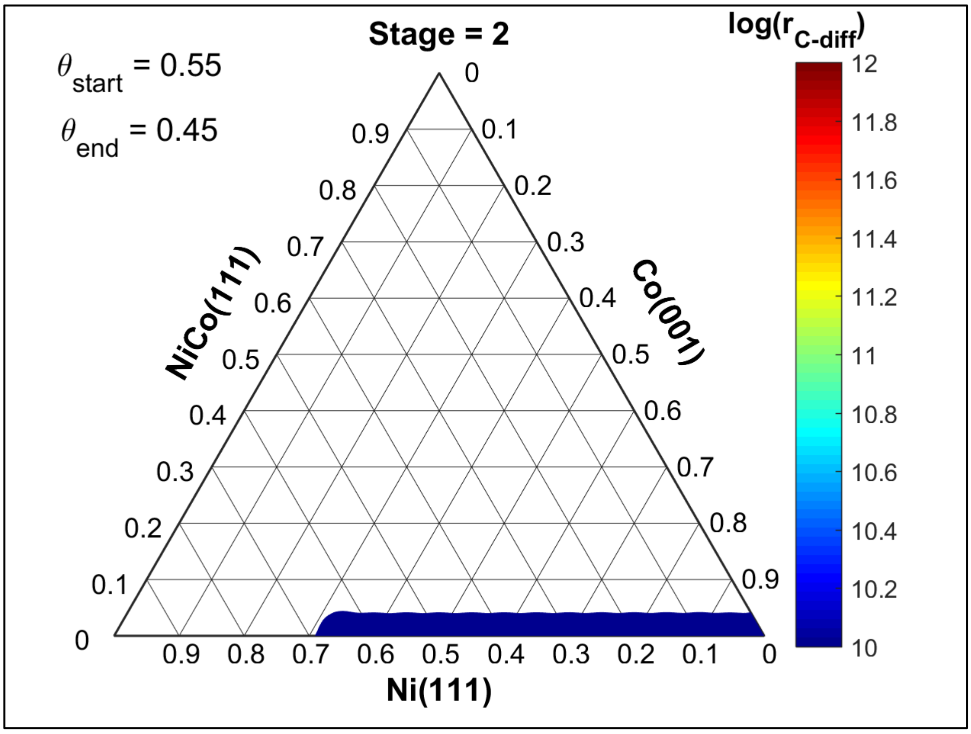

Figure S 28 TCP coke diffusion of the 2nd stage at reaction coordinate 24

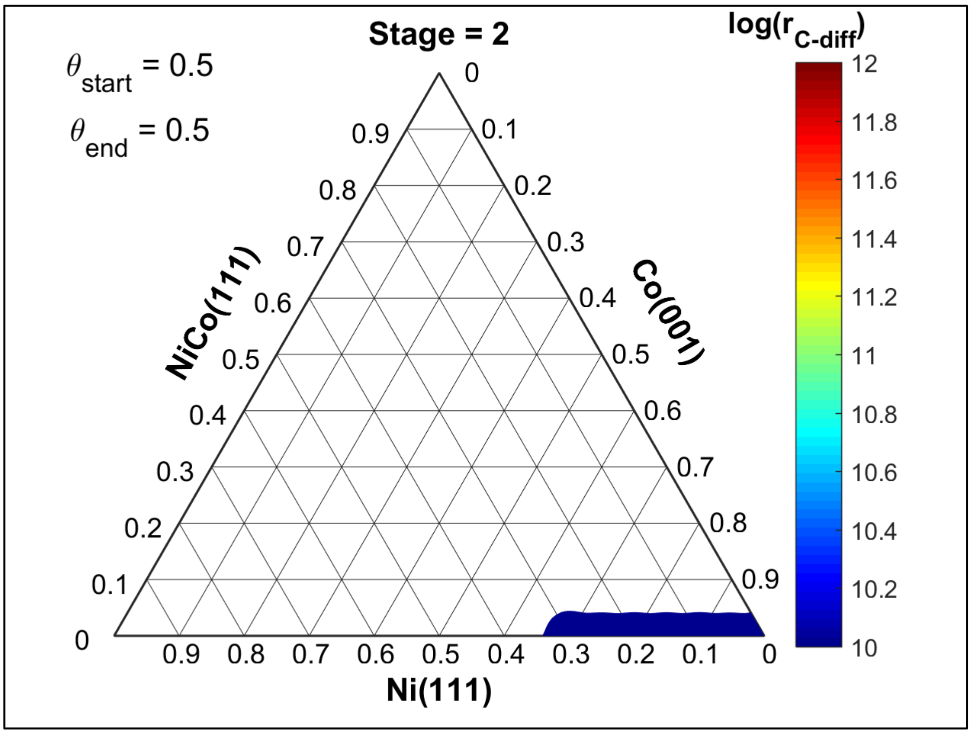

Figure S 29 TCP coke diffusion of the 2nd stage at reaction coordinate 25

3rd Stage Carbon Diffusion

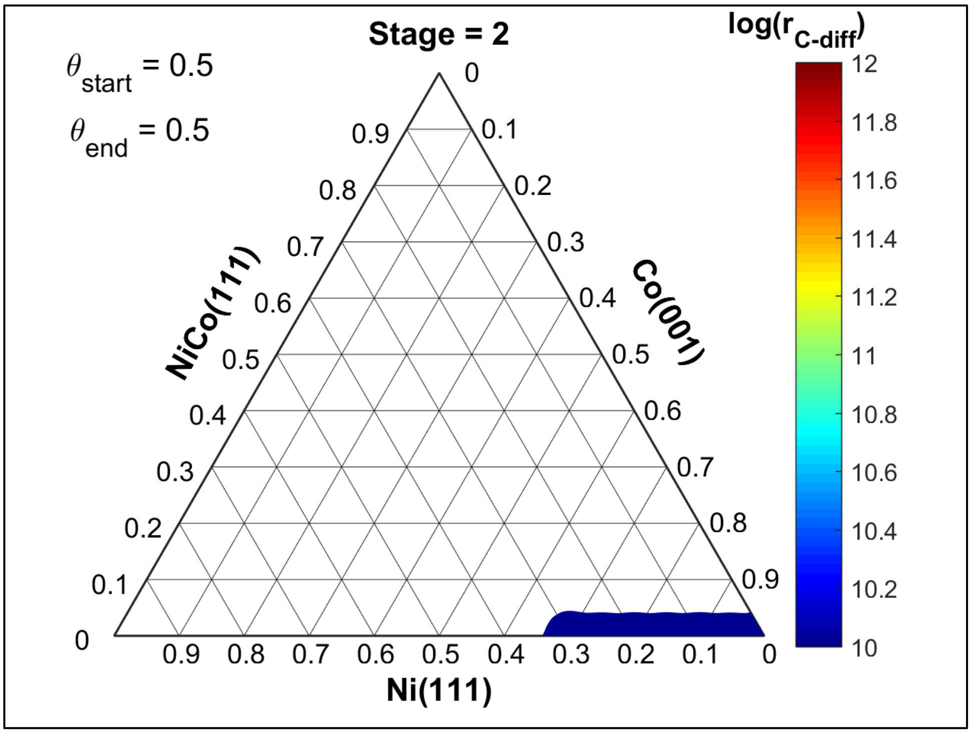

Figure S 30 TCP coke diffusion of the 3rd stage at reaction coordinate 25

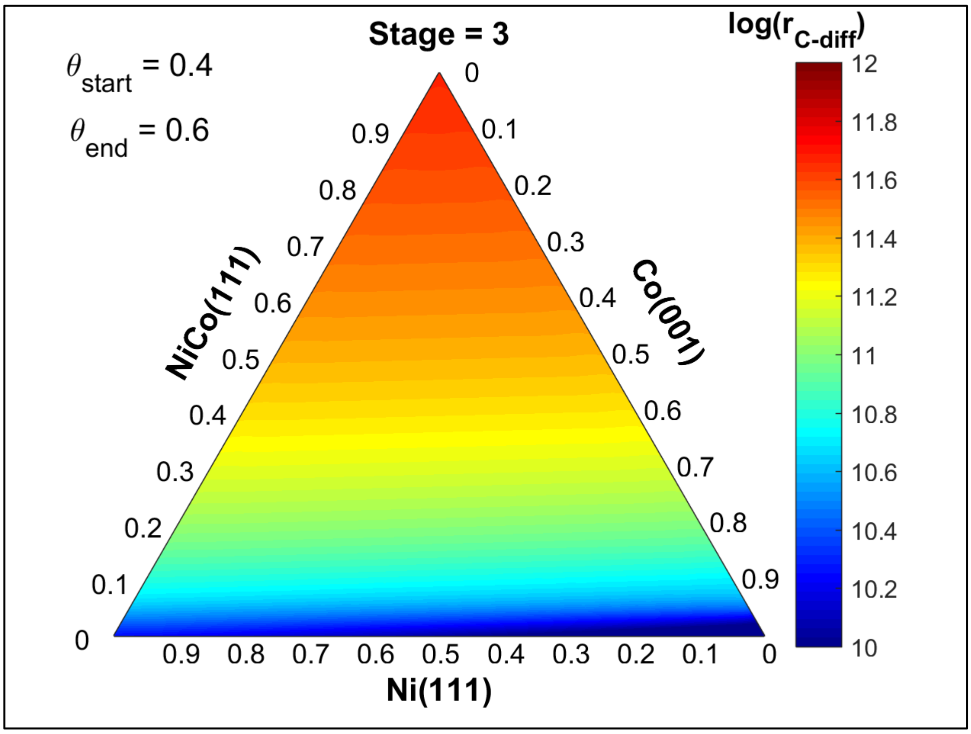

Figure S 31 TCP coke diffusion of the 3rd stage at reaction coordinate 26

3rd Stage Carbon Diffusion (continued)

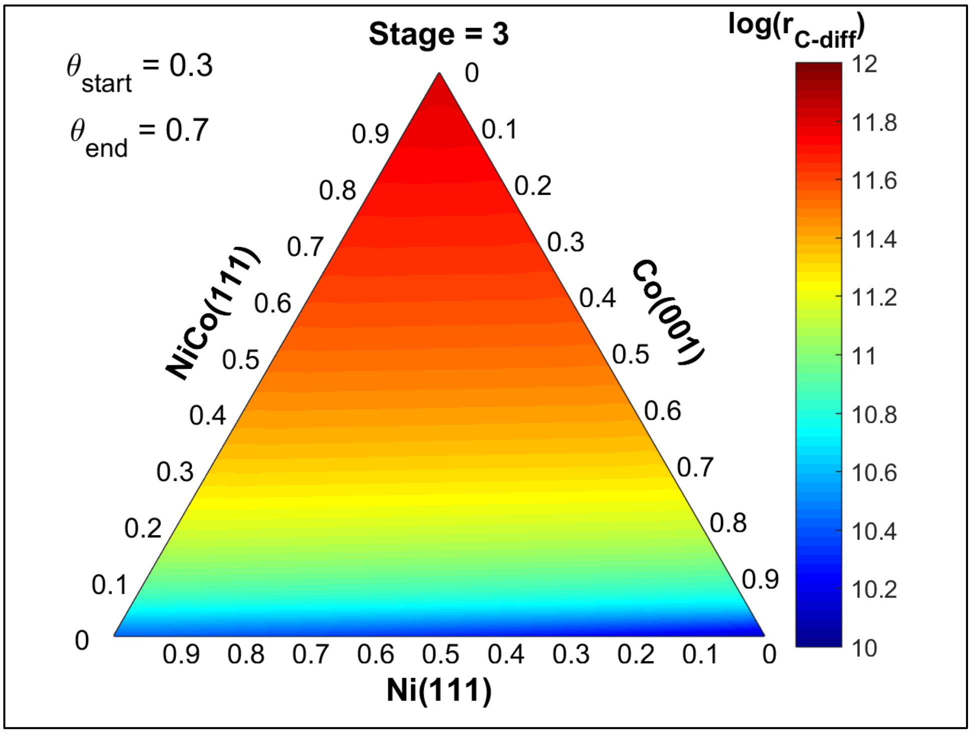

Figure S 32 TCP coke diffusion of the 3rd stage at reaction coordinate 27

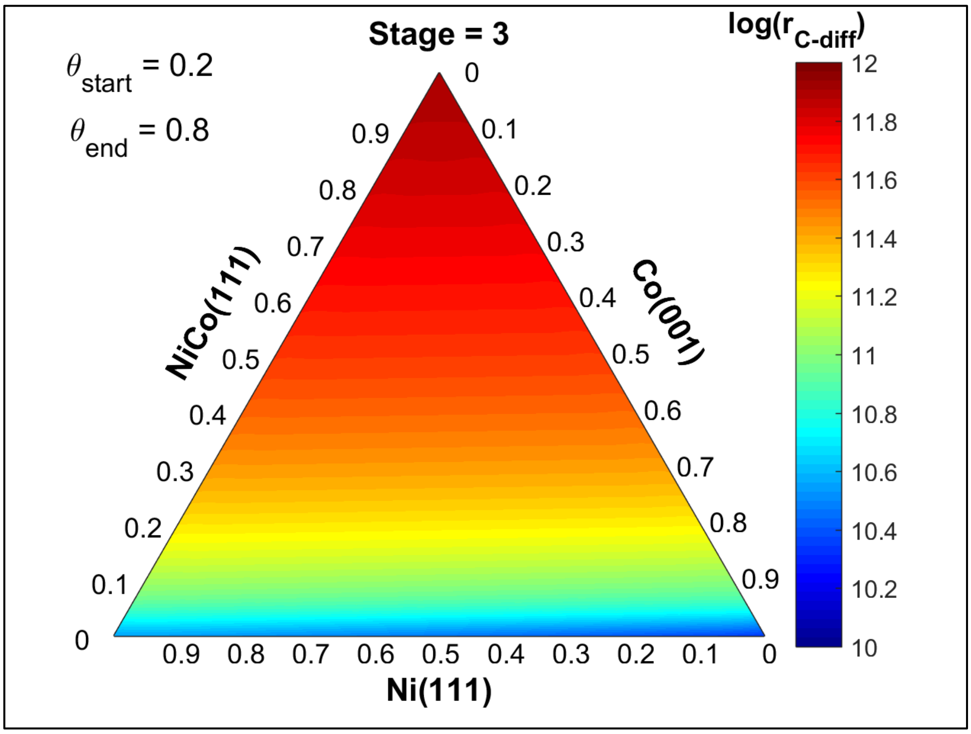

Figure S 33 TCP coke diffusion of the 3rd stage at reaction coordinate 28

<u>**3rd Stage Carbon Diffusion (continued)**</u>

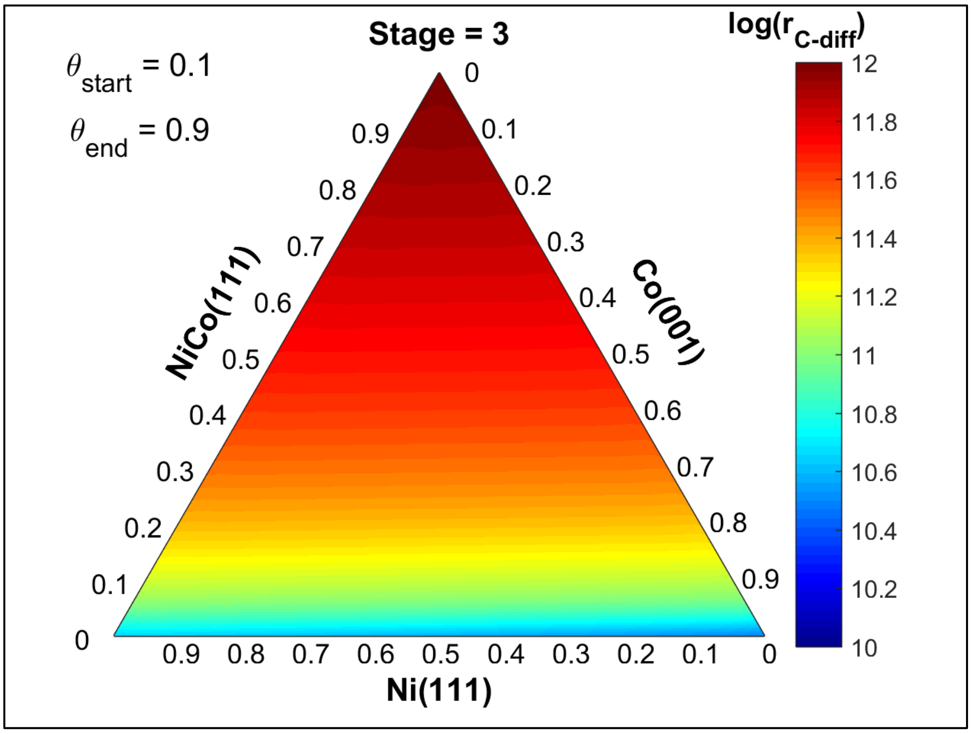

Figure S 34 TCP coke diffusion of the 3rd stage at reaction coordinate 29

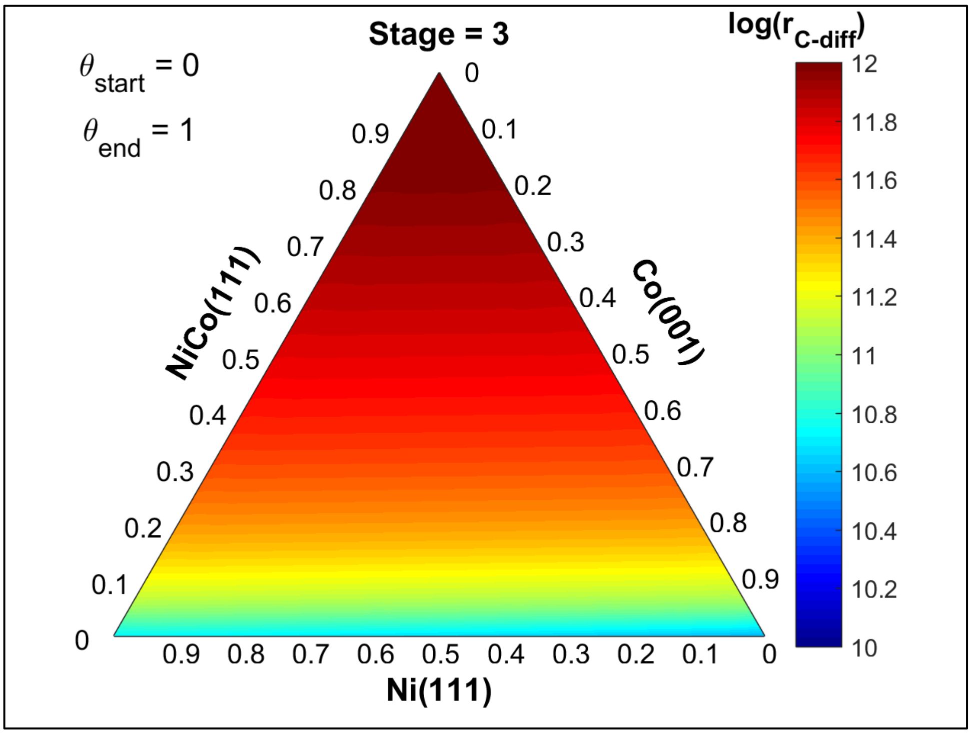

Figure S 35 TCP coke diffusion of the 3rd stage at reaction coordinate 30
